# Interventions to prevent alcohol use: systematic review of economic evaluations

**DOI:** 10.1192/bjo.2023.81

**Published:** 2023-06-27

**Authors:** Long Khanh-Dao Le, Jan Faller, Mary Lou Chatterton, Joahna Kevin Perez, Oxana Chiotelis, Huong Ngoc Quynh Tran, Marufa Sultana, Natasha Hall, Yong Yi Lee, Cath Chapman, Nicola Newton, Tim Slade, Matt Sunderland, Maree Teesson, Cathrine Mihalopoulos

**Affiliations:** PhD, Health Economics Group, School of Public Health and Preventive Medicine, Monash University, Australia; MHE, Health Economics Group, School of Public Health and Preventive Medicine, Monash University, Australia; PharmD, Health Economics Group, School of Public Health and Preventive Medicine, Monash University, Australia; MHE, Deakin Health Economics, Institute for Health Transformation, School of Health and Social Development, Deakin University, Australia; PhD, Deakin Health Economics, Institute for Health Transformation, School of Health and Social Development, Deakin University, Australia; PhD, Health Economics Group, School of Public Health and Preventive Medicine, Monash University, Australia; School of Public Health, The University of Queensland, Australia; and Policy and Epidemiology Group, Queensland Centre for Mental Health Research, Australia; PhD, Matilda Centre for Research in Mental Health and Substance Use, The University of Sydney, Australia

**Keywords:** Economic evaluation, systematic review, alcohol consumption, public health, return on investment

## Abstract

**Background:**

Alcohol use is a leading risk factor for death and disability worldwide.

**Aims:**

We conducted a systematic review on the cost-effectiveness evidence for interventions to prevent alcohol use across the lifespan.

**Method:**

Electronic databases (EMBASE, Medline, PsycINFO, CINAHL and EconLit) were searched for full economic evaluations and return-on-investment studies of alcohol prevention interventions published up to May 2021. The methods and results of included studies were evaluated with narrative synthesis, and study quality was assessed by the Drummond ten-point checklist.

**Results:**

A total of 69 studies met the inclusion criteria for a full economic evaluation or return-on-investment study. Most studies targeted adults or a combination of age groups, seven studies comprised children/adolescents and one involved older adults. Half of the studies found that alcohol prevention interventions are cost-saving (i.e. more effective and less costly than the comparator). This was especially true for universal prevention interventions designed to restrict exposure to alcohol through taxation or advertising bans; and selective/indicated prevention interventions, which involve screening with or without brief intervention for at-risk adults. School-based interventions combined with parent/carer interventions were cost-effective in preventing alcohol use among those aged under 18 years. No interventions were cost-effective for preventing alcohol use in older adults.

**Conclusions:**

Alcohol prevention interventions show promising evidence of cost-effectiveness. Further economic analyses are needed to facilitate policy-making in low- and middle-income countries, and among child, adolescent and older adult populations.

Alcohol use is a leading risk factor for death and disability worldwide, especially in young adults.^1^ The Global Burden of Disease study found that alcohol use is associated with substantial health loss, particularly in males.^1^ Importantly, the attributable burden of alcohol use increases monotonically with increasing alcohol consumption. Addressing alcohol-related harms is therefore a global public health priority.^2^ There are a variety of interventions designed to prevent alcohol use at the population level (i.e. upstream interventions, such as tax increases or advertising bans) and the individual level (i.e. downstream interventions, such as school-based interventions). To facilitate successful and sustainable scale-up of effective interventions and innovative service delivery strategies, decision makers require evidence on an intervention's cost and cost-effectiveness in addition to its effect on alcohol use and associated harms. Evaluating costs alongside the health effects of alcohol prevention and control strategies is required to determine their value-for-money credentials.

The burden of alcohol use disorders is exacerbated by its comorbidity with other substance use and mental health disorders. For example, a third of adults with opioid use disorder have an alcohol use disorder.^3^ Depression and anxiety are also most commonly associated with alcohol,^4^ with a third of people living in the UK reporting having both a psychiatric disorder and a comorbid alcohol use disorder.^4,5^

A previous review has identified 27 studies published between 2006 and 2016 that have examined economic evaluations of alcohol prevention interventions.^6^ Over half of the studies adopted a healthcare perspective, evaluating interventions over a 5-year time horizon. Most studies analysed healthcare costs, as well as costs attributable to government, social care, criminal justice, law enforcement and individual out-of-pocket payments. The studies evaluated a range of interventions, with the most common interventions comprising screening and brief interventions (SBIs), followed by upstream interventions such as tax increases, advertising restrictions and limiting retail sales. Only two school-based interventions were identified. However, this review primarily focused on economic evaluations of public health interventions and identifying methodological issues, rather than interpreting the cost-effectiveness results of broad preventive interventions for alcohol use in decision-making contexts. The evidence of economic benefit has grown rapidly since the previous review, necessitating an update. Importantly, there is also increasing evidence of economic evaluations targeting multiple health-related risk factors, including alcohol. Evidence on multifactorial prevention interventions were not included in the previous review.

## Study aims

This study aims to conduct a systematic review of the evidence for the cost-effectiveness of interventions to prevent alcohol use across the lifespan. This review used narrative synthesis to evaluate the methods of published economic evaluations and the quality of the literature. A key focus of this review was to summarise the cost-effectiveness evidence for alcohol prevention interventions and to identify knowledge gaps, challenges and opportunities for future research. Alcohol use often co-occurs with other substance use and mental/physical health conditions. As such, this review also evaluated studies assessing the cost-effectiveness of preventive interventions targeting multiple health-related risk factors alongside alcohol use.

## Method

### Search strategy

The current review adhered to the Preferred Reporting Items for Systematic Reviews and Meta-Analysis (PRISMA) guidelines^7^ and was registered on the International Prospective Register of Systematic Reviews Databases (PROSPERO; identifier CRD42020147386). The protocol was amended to include additional researchers and selecting on of the Drummond checklist as the tool for quality assessment. Searches were done to identify journal articles through electronic databases hosted on the EBSCOhost platform (i.e. EMBASE, Medline, CINAHL, PsycINFO and EconLit libraries) on 1 August 2019; with an updated done on 5 May 2021. The search strategy included economic evaluation terms; prevention or treatment terms; and terms related to alcohol, smoking, illicit drug use and substance use disorders. No date restrictions were applied during literature retrieval. Grey literature were excluded to narrow the focus on rigorous, peer-reviewed evidence. Unlike pharmaceutical products, many mental health prevention and treatment interventions are not subject to formal health technology assessment requirements. Manual searches were also conducted with the Tufts Cost-Effectiveness Analysis registry, a comprehensive database containing over 10 000 cost-effectiveness studies.^8^ Further details of the search strategy are presented in the Supplementary Material available at https://doi.org/10.1192/bjo.2023.81.

### Study selection

All citations were imported into a web-based systematic review software, Covidence (Veritas Health Innovation, Melbourne, Australia; www.covidence.org), which facilitated the identification and removal of duplicates. Title and abstract screening, full-text screening, data extraction and quality assessment were done independently by any two reviewers (J.F., L.K.-D.L., M.L.C., J.K.P., O.C., H.N.Q.T., M. Sultana, N.H.). Disagreements and discrepancies were resolved by a third reviewer (L.K.-D.L., M.L.C.). Studies were only included if they were full economic evaluations that compared two or more interventions in terms of their costs and outcomes.

Different economic evaluation frameworks can be used to assess the cost-effectiveness of healthcare interventions and programmes. Three commonly used frameworks include cost-effectiveness analysis (CEA), cost–utility analysis (CUA) and cost–benefit analysis (CBA). All of these frameworks measure costs in monetary terms, but differ in how outcomes are measured. For instance, outcomes are measured in CEA by using clinically meaningful units, e.g. the proportion who use alcohol, point improvements on a scale of alcohol-associated harms. The main units of outcome in CUA are generic health indices that combine measures of health-related quality of life (morbidity) and the length of life (mortality). Quality-adjusted life-years (QALYs) and disability-adjusted life-years (DALYs) are both commonly used generic health indices. In CBA, the most widely used framework beyond the health sector, all outcomes are valued in monetary terms. It follows that CBA necessitates the monetary valuation of health-related outcomes. Return-on-investment (ROI) analysis is also a commonly used partial economic evaluation framework that was included in this review. ROIs are typically a reduced form of CBA, where only the costs and cost offsets that can be attributed to healthcare interventions or programmes are considered compared with CBA, which often evaluates a wider set of health and non-health outcomes.

Economic evaluation studies can take the form of trial-based economic evaluations where the economic evaluation is conducted alongside a clinical trial. Alternatively, model-based economic evaluations synthesise multiple data sources to simulate the costs and outcomes that would occur under a scenario where an intervention is implemented versus some counterfactual scenario. All four economic evaluation frameworks (CEA, CUA, CBA and ROI) were included in this systematic review. Partial economic evaluations, cost studies, reviews, expert opinions, qualitative studies, conference papers, dissertations, book chapters and articles not in English were excluded. Studies were classified as alcohol prevention if they evaluated interventions focused on the prevention of alcohol use or the reduction of excessive alcohol use. Studies that targeted either the general population or the at-risk drinking population were included.

The mental health intervention spectrum described by Mrazek and Haggerty was used to classify prevention interventions into three types: universal, selective and indicated prevention.^9^ Universal prevention interventions target the whole population (e.g. school-based prevention). Selective prevention interventions target a subgroup of the population who are at risk for harmful alcohol use and/or binge drinking. Indicated prevention interventions target people who binge drink and/or consume harmful levels of alcohol, but do not have an alcohol use disorder or alcohol dependence. Studies were excluded if they included treatment interventions that target people diagnosed with an alcohol use disorder or alcohol dependence.

In summary, study inclusion criteria were full economic evaluations (e.g. CEA, CBA and CUA) or ROI studies aimed at prevention of alcohol use or reduction of excessive alcohol use. Exclusion criteria were partial economic evaluations and cost studies, reviews, expert opinions, qualitative studies, conference papers, dissertations, book chapters or artiwcles not in English.

### Data extraction

This study used a data extraction framework that was adapted from several previous reviews of economic evaluations and the review guideline developed by the Joanna Briggs Institute (JBI).^10,11^ Data extraction was completed in Microsoft Excel version 15.0 for Windows and independently performed by any two reviewers (J.F., L.K.-D.L., M.L.C., J.K.P., O.C., H.N.Q.T., M. Sultana, N.H.). Any discrepancies in data extraction were resolved by a third reviewer (J.F., J.K.P.), who was not involved in the initial extraction. Data were extracted on the target population, intervention(s) and comparator, economic evaluation framework, study design, perspective, time horizon, reference year, discount rates, currency, cost categories, outcomes measured and cost-effectiveness findings. There were no data extraction issues that warranted contacting the authors of included studies. However, if studies did not report an economic reference year, then it was assumed that the reference year was 2 years before the year of publication. To allow comparisons of value across studies, the reported intervention costs and ratios were converted into 2019 US dollars, using the EPPI-Centre cost conversion online tool.^12^

### Synthesis of study findings

Results were presented for the following age groups: children and adolescents (<18 years), adults (18–65 years) and older adults (>65 years). A meta-analysis was not conducted because of the substantive heterogeneity observed between studies in relation to the population, intervention, comparator, outcome and economic evaluation frameworks. We employed narrative synthesis together with a dominance ranking framework to synthesise study methods and findings. The dominance ranking framework presents the distribution of interventions across three decision criteria (i.e. favour, unclear decision or reject an intervention). This framework was adapted from the guideline developed by the JBI.^10^ Two reviewers (J.F., L.K.-D.L.) conducted the dominance framework classification. Dominance ranking was based on the results reported by the studies, and traffic light colour coding was used to indicate implications for decision makers. ‘Red’ signifies study results where routine adoption of the intervention is likely to be less favoured or rejected by decision makers (i.e. costs are higher and the intervention is less effective). ‘Green’ denotes study results that suggest an intervention is potentially very acceptable or favourable to decision makers (i.e. has better health outcomes and lower costs). ‘Yellow’ signifies study results that do not provide a clear-cut decision for decision makers (i.e. the intervention is ‘more effective and more costly’ or it is ‘less effective and less costly’). In this case, some form of financial or clinical trade-off is required. Willingness-to-pay thresholds can be used here to determine whether the intervention is cost-effective and represents value for money.

### Quality assessment

Reporting and quality assessment was completed with the Drummond ten-point checklist.^13^ Despite planning to use the Quality of Health Economic Studies (QHES) tool, we ultimately opted to use the Drummond ten-point checklist because it can be applied to both trial- and model-based economic evaluations (the QHES is only applicable to model-based evaluations). Two independent reviewers were involved in the quality assessment of included studies. Conflicts were resolved by a third reviewer (J.F., L.K.-D.L., M.L.C.). There are 33 sub-items attached to the ten overarching Drummond criteria, which can be answered as ‘yes’, ‘no’ and ‘cannot tell’.^13^ Items that were relevant but did not have sufficient information to judge ‘yes’ or ‘no’ were marked with ‘cannot tell’. Reporting and quality assessment were completed in Microsoft Excel, with two reviewers independently assessing the quality of included studies. To limit inconsistencies in assessment, the authors met to discuss and assess two identified studies (a trial-based and a model-based economic evaluation). An average score was calculated to gauge the quality of the studies. ‘Yes’ answers were assigned a score of 1; ‘no’ answers were assigned a score of 0 and ‘cannot tell’ were assigned a score of 0.5. Studies that scored at least 9 were considered of good quality, studies that scored 6 to <9 were considered of fair quality,^14^ and studies scoring <6 were deemed poor quality, but were still presented to show the entirety of the available evidence. Quality assessment was also discussed narratively to describe the characteristics of identified studies. Post-quality assessment internal consistency was measured with the Kuder–Richardson Formula 20 (KR20). For each study, a binary entry (0 for conflict or 1 for agreement) was used to represent independent quality assessment. A KR20 coefficient ranges from 0 to 1, with a score closer to 1 indicating high internal consistency.

## Results

**Fig. 1:**
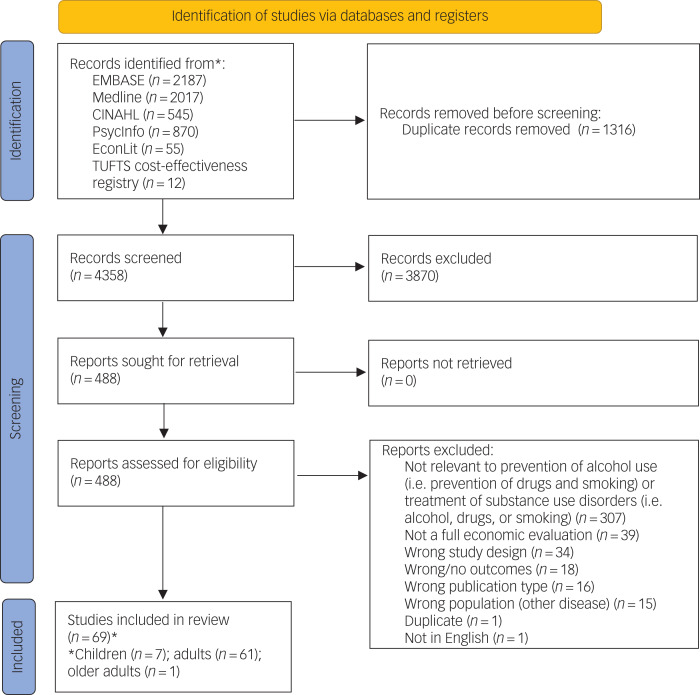
Preferred Reporting Items for Systematic Reviews and Meta-Analyses (PRISMA) diagram.

A total of 5674 articles were identified during the literature search. After removing duplicates and title and abstract screening, 488 articles remained for full-text deliberation. There were 364 articles remaining after full-text screening. Of these, 57 studies met the inclusion criteria for the prevention of alcohol use (Fig. 1). The main reasons for exclusion were as follows: being outside the scope of the review (e.g. prevention of other substances or substance use disorder treatments), did not meet the criteria of a full economic evaluation, incorrect disease population, incorrect study designs or publication type, and wrong or no outcomes. Hand searching further identified 12 studies. The reasons for missing out on the 12 articles identified through hand searching were exclusion through screening (*n* = 2) and from combining the concepts within the search strategy (*n* = 10). There were 34 model-based evaluation studies, 28 trial-based evaluations and seven studies that included both model- and trial-based economic evaluations. The economic evaluation frameworks comprised CEA (*n* = 21), CUA (*n* = 18), CBA (*n* = 9) and ROI (*n* = 4). There were also studies that used multiple economic evaluation frameworks, including CBA plus CEA (*n* = 4) and CEA plus CUA (*n* = 13). Sixty-one studies were for the general or adult population, and the remaining studies involved children (*n* = 7) or older adults (*n* = 1). Further details of included economic evaluations are found in Table 1.

**Table 1:** Data extraction of included economic evaluations

Author	Target population	Intervention(s) and comparator	Economic evaluation framework, study design	Perspective, time horizon, reference year, discount rates, currency	Cost categories	Outcomes measured	Cost-effectiveness findings (in 2019 US dollars)^a^	Quality score^b^, verdict
Children and adolescents								
Agus et al (2019),^16^ UK	Students in school year 8/S1 (aged 11–12) (selective)	Intervention: The Steps Towards Alcohol Misuse Prevention Program (STAMPP) – a classroom-based alcohol education curricula, combined with a brief alcohol intervention for parents/carers (*n* = 6379) Comparator: education as normal (*n* = 6359)	CEA RCT (*n* = 12 738)	Public sector, 33 months, 2013–2014, 3.5%, GBP	Costs to local authorities, national health service, personal social services and criminal justice services Education: school nurse, school counsellor/guidance teacher, intervention teacher, education psychologist, education welfare officer/home school liaison officer Health: GP surgery visit, GP out of hours, nurse (other than school nurse), hospital appointment, accident and emergency, overnight hospital stay, psychologist, counsellor (other than at school), social worker, telephone helpline Criminal justice: youth justice service, police service	Cost per young person experiencing heavy episodic drinking avoided because of STAMPP, at 33 months from baseline	£–17.19 ($27) per 0.08 heavy drinking episode avoided (previous 30 days) STAMPP can be said to dominate usual education; however, since the difference in costs was not statistically different, only weak dominance can be claimed	8.5, fair
Deluca et al (2021),^17^ UK	Low-risk adolescents (14–17 years) (selective)	Screening control (*n* = 304); one face-to-face session of PFBA (*n* = 285); PFBA plus an eBI on smartphone or web (*n* = 294)	CUA RCT (*n* = 883)	Societal and NHS + PSS, 12 months, 2014, no discounting needed, GBP	Treatment and NHS and non-NHS costs (primary and secondary care, specialist health services, social services and the criminal justice system)	QALY	Screening dominates eBI; PFBA ICERs £131 000 ($204 027) per QALY gained (societal) and £121 000 ($188 453) per QALY gained (NHS + PSS). Based on WTP of £30 000 ($46 724) per QALY, PFBA is not cost-effective	8.5, fair
Downs and Klein (1995)^21^, USA	Adolescents and youths who were identified as high risk of the two risky behaviours, alcohol misuse and unsafe sexual activity (selective)	Intervention: a programme of screening visits for all adolescents and counselling visits for youth identified as high risk Comparator: standard practice	CEA model	Societal, 5 years, not available, no discount, USD	Preventive programme cost per teen at low risk, preventive programme cost per teen at high risk, societal cost of a motor vehicle crash, cost of treating a case of STD, cost of managing a case of HIV, cost of managing a teen pregnancy	Life saved	5% efficacy: $14 699 ($23 961) per STD case prevented; $15 312 ($24 960) per pregnancy prevented; $12 070 ($19 675) per motor vehicle crash prevented; $12 million ($19.56 million) to prevent a death owing to motor vehicle crash; $490 000 ($798 738) per case of HIV prevented; $4580 ($7466) to prevent any one of the adverse outcomes considered 5.6% efficacy: breakeven point. Any higher efficacy will result in cost-savings. At $600 000 ($978 047) WTP, intervention is cost-effective	5, poor
Drost et al (2016),^18^ The Netherlands	Dutch adolescents (aged 15–19 years) attending school, participants included students at schools of higher secondary education, lower secondary education and lower vocational training (selective)	Intervention: web-based computer-tailored intervention for reducing alcohol use and binge drinking by adolescents, a game with tailored feedback on alcohol awareness (*n* = 1538) Comparator: care as usual (*n* = 955)	CEA RCT (*n* = 2493)	Healthcare and societal, 4 months, 2014, not applicable, Euro	Intervention costs: hosting costs for the website, tailored feedback software, participants’ time investments Healthcare costs: health services used, including contacts with the general practitioner emergency care, hospital stays, ambulance rides and mental health services Intersectoral costs: school absenteeism and contacts with an attendance officer (education), work absenteeism (labour and social security), failing to perform household and other activities, contacts with youth and family centre and family care (household and leisure) and contacts with (youth) police services, court proceedings and child (health) protection services (criminal justice system) Costs of substance use: packs of cigarettes, use of soft drugs and use of hard drugs	Reduction of one glass of alcohol per week and one binge drinking occasion per 30 days	Healthcare perspective: €40 ($53) to €79 ($106) per reduction of one glass of alcohol per week and one binge drinking occasion per 30 days Societal perspective: €62 ($83) to €144 ($192) per reduction of one glass of alcohol per week and one binge drinking occasion per 30 days	8.5, fair
Ingels et al (2013),^20^ USA	African American adolescents and their primary caregivers (selective)	The Strong African American Families-Teen (SAAF-T) programme versus attention-control intervention	CEA RCT (*n* = 473)	Societal, 15–18 months, 2009, not reported, USD	Intervention cost	Episodes of alcohol use prevented	Compared with the attention control, the SAAF-T programme cost $50 ($59) per reduction in an alcohol use episode and $123 ($145) per reduced episode of binge drinking	8, fair
Kuklinski et al (2015),^19^ USA	Youth 12 grade students (selective)	Communities That Care (CTC) prevention programme compared with control group	CBA RCT (*n* = 4,407)	Participants, taxpayers, other beneficiaries, 5 years, 2011, 3%, USD	(1) community coalition; (2) intervention programmes; (3) training, technical assistance and implementation monitoring and (4) other costs	Monetary benefits from preventing delinquency, alcohol use and smoking	CTC cost:benefit = $4477:$556 ($5126:$637) per youth for 5 years Net present benefit: $3920 ($4488); benefit–cost ratio was $8.22 per dollar invested. CTC is cost-beneficial in sensitivity analysis	10, good
Spoth et al (2002),^15^ USA	Families of sixth graders (selective)	Iowa Strengthening Families Program (ISFP) – parenting skills with child involvement (*n* = 162); Preparing for the Drug-Free Years programme (PDFY) – more on parenting skills (*n* = 153); and minimal contact (*n* = 163)	CBA, CEA Economic evaluation alongside RCT modelling (piggyback model) (*n* = 478)	Societal, lifetime time horizon, 1992, 3%, USD	Intervention costs, incentives, childcare, parent travel	Cases prevented, costs	$12 459 ($20 790) per case prevented and $9.60 saved for every dollar invested (ISFF). $20 439 ($34 106) per case prevented and $5.85 saved for every dollar invested (PDFY)	9, good
Adults								
Angus et al (2014),^52^ Italy	Italian population, both males and females, aged 16 to ≥75 years (universal)	Intervention 1: screening and brief intervention at next GP registration Intervention 2: screening and brief intervention at next GP consultation Comparator: ‘do nothing’ scenario	CUA model	Healthcare, 30 years, 2008, 3%, Euro	Healthcare costs: in-patient, out-patient and accident and emergency visits and other costs Cost of screening and brief intervention: cost of briefing materials provided to the patient and the cost of the GP's time, overheads and other related costs	QALY	Screening at next GP registration: general population: €550/QALY ($851/QALY); men-only population: cost-saving; women only: €3100/QALY ($4797/QALY); SA 0% discount rate: cost-saving; SA 5% discount rate: €1200/QALY ($1857/QALY) Screening at next GP consultation: €590/QALY ($913/QALY); SA 0% discount rate: €60/QALY ($93/QALY); SA 5% discount rate: €1100/QALY ($1702/QALY)	9, good
Angus et al (2017),^53^ Europe	General population -heavy drinkers (Europe with country breakdown) (universal)	No screening and brief intervention versus screening and brief intervention delivery scenarios	CUA Economic modelling, Sheffield model	Healthcare system, 10-year time horizon, 2013, 3.5%, Euro	GP, hospital and programme costs	QALY	Using baseline factor values: cost-effective for all four countries England: €7574/QALY ($13 053/QALY) Italy: €1168/QALY ($1807/QALY) The Netherlands: €903/QALY ($1260) Poland: €3021/QALY ($1762) Using collected factor values: Cost-saving: Austria, Belgium, Cyprus, Denmark, France, Greece, Ireland, Luxembourg, Malta, The Netherlands, Portugal, Spain, Swede, England Cost-effective: Czech Republic, Finland, Germany, Hungary, Italy, Latvia, Lithuania, Poland, Slovakia, Slovenia Not cost-effective: Bulgaria, Croatia, Estonia, Romania	8.5, fair
Babor et al (2006),^40^ USA	Patients screening positive for at-risk drinking in managed healthcare organisations (selective)	Intervention 1: brief intervention delivered by licensed practitioners (P condition) Intervention 2: brief intervention delivered by mid-level professional specialists (nurses) (S condition) Comparator: usual care	CEA pre–post, quasi-experimental, multi-site evaluation (*n* = 1329)	Not available, 3 and 12 months, not available	Ongoing implementation cost: labour costs of the health appraisal, AUDIT screening and delivery of the intervention, space costs for intervention activities Production costs of health appraisal, screening and intervention materials used at each site (Media costs include reproduction costs of screening and patient education materials)	Primary outcome: number of drinks per week Secondary outcomes: frequency of heavy drinking, SF-12 measures of quality of life	Incremental cost of SBI per patient for both screening and intervention is estimated to be $3.53 ($5) in the S condition and $4.87 ($6) in the P condition	8.5, fair
Barbosa et al (2015),^54^ USA	9835 SBIRT screen-positive patients, 39% of patients consumed drugs in the past 30 days at baseline, 29% consumed both drugs and alcohol, 74% of people using drugs also used alcohol (indicated)	Intervention: alcohol screening, brief intervention and referral to treatment (SBIRT) in different settings, e.g. emergency department or out-patient setting	CUA model	Provider and societal, 6 months, 2011, not reported, USD	Provider perspective: costs to those delivering services Societal perspective: costs to society: healthcare utilisation, including the provider of SBIRT, criminal activity, automobile accidents and lost income	The proportion of patients not drinking above threshold levels at follow-up The proportion of patients transitioning from above threshold levels at baseline to abstinent or below threshold levels at follow-up 3. QALY	For both a provider and social perspective, SBIRT in the emergency department setting dominates the out-patient setting	9, good
Barbosa et al (2017),^41^ USA	Patients had 6-month follow-up data in the records in Government Performance Results Act (GPRA) survey (universal)	Intervention: brief intervention (*n* = 878) Comparator: brief treatment (*n* = 98) within SBIRT programmes	CEA quasi-experimental design (*n* = 976)	Provider, not available, 2012, not available, USD	Labour costs, material costs and space costs	Proportion using alcohol Proportion using alcohol to intoxication Days of alcohol use Days of alcohol use to intoxication Proportion using drugs Days using drugs	Brief treatment: $8.9 ($10) per one percentage point reduction in the probability of using any alcohol	7, fair
Barrett et al (2006),^24^ UK	Alcohol misusing patients (indicated)	Brief intervention (*n* = 287) versus information only (*n* = 312)	CEA RCT (*n* = 599)	A broad cost perspective, 12 months, in 2001/02, GBP	Health and social services costs, criminal justice costs and productivity losses	The mean number of alcohol units consumed per week	ICER of £22 ($44) per unit reduction in the amount of alcohol consumed per week	7.5, fair
Blankers et al (2012),^34^ The Netherlands	Participants aged between 18 and 65 years, live in The Netherlands with healthcare insurance coverage, have internet access at home, score above 8 on the AUDIT, report a week consumption of >14 standard drinking units and provide informed consent (indicated)	Intervention: internet-based intervention for harmful use of alcohol through the assessment of the incremental cost-effectiveness of intervention-based therapy (*n* = 68) Comparator: internet-based self-help (*n* = 68)	CEA, CUA RCT (*n* = 136)	Societal, 6 months Sensitivity analysis: healthcare provider, 2010, not applicable, Euros	Software development costs, information and computer technology service costs, overhead costs and IT-only therapist-related costs Productivity losses: absenteeism and presenteeism Additional societal costs, e.g. additional healthcare resource costs, and low-enforcement costs	Additional treatment responder QALY	Societal perspective: €3683 ($5328)/additional treatment responder €14 710 ($20 287)/QALY Healthcare provider perspective: € 1157 ($1596)/additional treatment responder €4693 ($6472)/QALY	8, fair
Brennan et al (2014),^48^ UK	Adults and young people aged 16 or more, including subgroups of moderate, hazardous and harmful drinkers (universal)	Below-cost selling bans compared with minimum unit pricing	CEA, CUA Economic modelling, Sheffield model	National policy, 10 years, 2014–2015, 3.5%, GBP	Costs to the NHS	Mean consumption changes, expenditure and reductions in deaths, illnesses, admissions to hospital and QALY	45 p minimum unit price versus a ban on below-cost selling (24 200 *v.* 500) total QALY gained and healthcare cost-savings −£9.5 million *v*. −£417.2 million (–$14.73 million *v.* −$646.95 million)	5.5, poor
Byrnes et al (2010),^82^ Australia	General Australian population (universal)	Intervention: volumetric alcohol taxation, three scenarios: - No change in deadweight loss - No change in tax revenue - All alcoholic beverages taxed at the same rate as spirits Comparator: current policy	CUA Mathematical modelling	Healthcare, 20 years, 2003, 3%, AUD	Cost to government of implementing a volumetric tax Hospital and medical costs averted from reduced alcohol consumption	Change in alcohol consumption Tax revenue Health benefit	A volumetric tax that is deadweight loss neutral: Increase in taxation revenue: $492 million ($521 million) 2.77% reduction in annual consumption of pure alcohol Health gain: 21 000 DALYs A tax revenue neutral: 0.05% decrease in consumption A tax on all alcohol at a spirit's rate: 23.85% reduction in consumption Increase revenue: $3094 million ($3276 million)	8.5, fair
Cadilhac et al (2011),^72^ Australia	The general 2008 Australian population (universal)	Eliminating one of the six risk factors from the population, including tobacco use and alcohol consumption	CUA model	Societal, lifetime horizon, 2008, 3%, AUD	Productivity losses including household production and leisure time, health sector cost	DALY	The largest potential savings could be gained from reductions in alcohol consumption followed by reductions in tobacco smoking. We did not include intervention costs for this analysis, and we have assumed that effective interventions exist to achieve the target reduction in the prevalence of the risk factors	6.5, fair
Chisholm et al (2004),^56^ global	WHO regions: Africa, the Americas, Eastern Mediterranean, Europe, South-East Asia, Western Pacific (universal)	Brief intervention Law enforcement (e.g. random breath testing of drivers) Policy and legislative intervention, including taxes on alcohol sales, drink-driving laws, restricted licensing outlets and advertising control Mass media/awareness campaigns	CUA model	Societal, lifetime horizon, not available, 3%, international dollar	Administration, training and media, and patient-level costs, such as primary visits	DALY	The most effective and cost-effective intervention was taxation (more than 500 DALYs averted per 1 million population) Breath testing: range from dominated to $53–$2671 ($73–$3702) per DALY Restricted access: range from dominant to $74–$2942 ($103–$4078) per DALY Advertising ban: range from dominant to $87–$2131 ($121–$2954) per DALY Brief physician advice: range from dominant to dominated Highest tax plus advertising ban: range $79–$1745 ($110–$2419) per DALY Highest tax, advertising ban and brief advice: range $240–$2786 ($333–$3862) per DALY	9, good
Chisholm et al (2018),^55^ global	General population in 16 countries spanning low-, middle- and high-income settings across the world: China, Germany, Japan, Mexico, Russian Federation, South Africa, Thailand, Turkey, USA, Ethiopia, Guatemala, India, Nigeria, Philippines, Ukraine, Vietnam (universal)	Increase in excise taxes on alcoholic beverages Enactment and enforcement of bans or comprehensive restrictions on exposure to alcohol advertising (across multiple types of media) Enactment and enforcement of restrictions on the physical availability of retailed alcohol (via reduced hours of sale) Enactment and enforcement of drink-driving laws and BAC limits (via sobriety checkpoints) Provision of brief psychosocial interventions for persons with hazardous and harmful alcohol use	CUA model	Not available, lifetime horizon, 2010, 3%, international dollar	For brief psychosocial interventions: contacts with primary healthcare (for screening, assessment, intervention and follow-up), out-patient, in-patient hospital care for a proportion of case For other (population-based) measures: human resources (e.g. administrators, lawyers), training (e.g. enforcement), meetings, mass media and law enforcement/inspection (including related equipment such as a handheld speed camera, breath alcohol analyser, traffic cones and police vehicle for roadside checkpoints)	Healthy life-years gained	Pricing policies and restrictions to alcohol availability and marketing continue to represent a highly cost-effective use of resources CER: Intervention 1: $22–$41 ($26–$48)/healthy life-year gained Intervention 2: $48–$120 ($56–$140)/healthy life-year gained Intervention 3: $77–$181 ($90–$212)/healthy life-year gained Intervention 4: $1454–$2979 ($1700–$3482)/healthy life-year gained Intervention 5: $143–$1434 ($167–$1676)/healthy life-year gained	6, fair
Cobiac et al (2009),^66^ Australia	The general 2003 Australian population (universal)	Eight interventions: volumetric taxation, advertising bans, an increase in minimum legal drinking age, licensing controls on operating hours, brief intervention (with and without general practitioner telemarketing and support), drink-driving campaigns, random breath testing and residential treatment for alcohol dependence (with and without naltrexone) Comparator: current practice	CUA model	Health sector, lifetime horizon, 2003, 3%, AUD	Government costs for materials and personnel associated with delivering and/or enforcing each intervention, and costs to patients associated with participation and time and travel to attend (e.g. GP brief intervention sessions), but excludes costs associated with alcohol-related crime and violence and any costs owing to lost productivity. Intervention start-up costs (e.g. costs of research and development of intervention material for brief intervention) are not included	DALY	All seven preventive interventions would be a cost-effective investment that could lead to substantial improvement in population health; only residential treatment is not cost-effective Two interventions stand out as being most effective and most cost-effective – changes to taxation and advertising bans Volumetric taxation: dominant Advertising ban: dominant Licensing controls: $3300 ($3495)/DALY averted Brief intervention: $6800 ($7201)/DALY averted Brief intervention, telemarketing and support: $10 000 ($10 589)/DALY averted Residential treatment: $190 000 ($201 200)/DALY averted Residential treatment and naltrexone: $120 000 ($127 074)/DALY averted Minimum legal drink age raised to 21: dominant Drink-driving mass media: $14 000 ($14 825)/DALY averted	6.5, fair
Coulton et al (2017),^32^ UK	Older hazardous alcohol users in primary care, patients ≥55 years, scoring ≥8 on the AUDIT (indicated)	Intervention: a stepped-care intervention: an initial 20-min of behavioural change counselling, with step 2 being three sessions of motivational enhancement therapy and step 3 being referral to local alcohol services (*n* = 266) Comparator: a minimal intervention of 5 min of brief advice (*n* = 263)	CUA RCT (*n* = 529)	NHS/personal social care, 1 year, 2009–2010, not applicable, GBP	Health services, other alcohol services outside the study, public and criminal justice services, training, supervision, management and overheads	Average drinks per day AUDIT-C Alcohol-related problems measured using the Drinking Problems Index Health-related quality of life measured using the SF-12	Stepped care is a dominant intervention when compared with minimal intervention in both 6- and 12-month time horizons	7.5, fair
Cowell et al (2012),^31^ USA	Heavy drinking among university freshmen (indicated)	Assessment only, motivational interviewing, feedback only and MIFB	CEA RCT (*n* = 727)	Intervention provider, 12 months, 2009, not applicable, USD	Fixed costs: original, replacement, total staff training, equipment costs. Variable costs: intervention cost (monitoring participant, preparing feedback report, conducting motivational interviewing)	Changes in average drinks per drinking occasion and number of heavy drinking occasions	ICER (MIFB) = $47.04 ($56)/average drinks per drinking occasion and $64.34 ($76)/heavy drinking days; motivational interviewing and feedback only are economically dominated by MIFB	8.5, fair
Cowell et al (2018),^78^ USA	Probationers in the USA (indicated)	Intervention 1: a computerised intervention, Motivational Assessment Program to Initiate Treatment (MAPIT), intervention delivered at the outset of probation. A two-session motivational computer-based intervention (*n* = 104) Intervention 2: face-to-face motivational interviewing, intervention delivered at the outset of probation. A two-session counsellor-delivered intervention (*n* = 103) Comparator: supervision as usual (*n* = 109)	CEA RCT (*n* = 316)	Probation system, 1 year, 2016, not applicable, USD	Development costs: software development cost Start-up costs: computers, printers and licenses for web hosting, message texting and the text-to-speech software, staff time for training and the amount and costs of purchased items Implementation costs: screening and assessment costs, oversight or deliver costs, scheduling costs, clinical supervision costs, space and material costs	Percentage point increase in the probability of initiating treatment or treatment initiation	Relative to supervision as usual, MAPIT costs $6.70 ($7) per percentage point increase in the probability of initiating treatment. Motivational interviewing is dominated by MAPIT	9, good
Crawford et al (2015),^28^ UK	Population aged 19 years or over who attended one of three sexual health clinics and were drinking excessively (selective)	Intervention: brief advice consisted of feedback on alcohol and health written information and an offer of an appointment with an alcohol health worker (*n* = 402) Comparator: control group – control participants received a leaflet on health and lifestyle (*n* = 400)	CUA RCT (*n* = 802)	NHS/personal social care, 6 months, 2010–2011, not applicable, GBP	Medication, hospital contacts and community health and social services	Mean weekly alcohol consumption during the previous 90 days measured 6 months after health-related QALY from EQ-5D	The cost-effectiveness acceptability curve demonstrates that there is no evidence that brief advice is cost-effective at any willingness to pay values for a QALY Intervention cost: £12.57 ($21) Intervention QALYs were 0.007 lower in among those allocated to brief advice	6, fair
Ditsuwan et al (2013),^45^ Thailand	General population (universal)	Current coverage of sobriety checkpoints and media campaigns, full coverage of sobriety checkpoints and media campaigns, and do nothing	CEA Economic modelling, decision tree	Health sector, lifetime time horizon, 2004, 3% discount rate, Thai Baht	Healthcare costs (fatality, disability, admission and non-admission related costs)	Percentage reduction in the burden of road traffic injury attributable to driving under the influence, DALY	Compared with ‘doing nothing’, all interventions were dominant because of cost-savings	9, good
Doran et al (2013),^49^ Australia	General population, 18 years and older (universal)	Doing nothing versus other scenarios Scenario 1: replace the wine equalisation tax on wine and cider with a volumetric excise rate equal to the current excise tax rate applicable to low-strength beer sold offsite Scenario 2: apply an excise tax rate to all beverages equal to a 10% increase in the current excise tax rate applicable to spirits and ready-to-drink products Scenario 3: apply an excise tax rate to all beverages, increasing it exponentially by 3.0% for every 1.0% increase in alcohol content above 3.2% Scenario 4: apply a two-tiered volumetric excise tax rate: the first tier applies to beer and wine and increases exponentially by 3.0% for every 1.0% increase in alcohol content above 3.2%; the second tier applies the current excise tax rate applicable to spirits and ready-to-drink beverages	CUA Economic modelling, multi-state, multiple-cohort life table model	Health sector, lifetime time horizon, 2009, 3%, AUD	Healthcare costs, taxation revenue	DALY	All interventions dominant compared with base scenario	6.5, fair
Fleming et al (2000),^29^ USA	Problem drinkers aged 18–65 years, men who consumed >14 drinks per week, women who consumed >11 drinks per week (indicated)	Brief physician advice (*n* = 392) and control group (*n* = 382)	CBA RCT (*n* = 774)	Societal, 1 year, 1993, not reported, USD	Healthcare (intervention) costs, patient opportunity costs (lost wages, transportation), society (alcohol-related accidents, legal events)	Alcohol use, emergency department visits, hospital days, legal events, motor vehicle accidents in monetary terms	The benefit:cost ratio was 5.6:1, or $56 263 in total benefit for every $10 000 invested	8.5, fair
Fleming et al (2002),^30^ USA	Problem drinkers aged 18–65 years, men who consumed >14 drinks per week, women who consumed >11 drinks per week (indicated)	Brief physician advice (*n* = 392) and control group (*n* = 382)	CBA RCT (*n* = 774)	Medical and societal, 48 months, 1993, not reported, USD	Intervention costs (clinic cost and patients cost)	Alcohol use, motor vehicle and legal events, injuries, healthcare utilisation, health status, and mortality in monetary terms	Benefit:cost ratio: 4.3:1 from a medical perspective; 39:1 from a societal perspective $43 000 reduction in future healthcare costs for every $10 000 invested in early intervention	7.5, fair
Galárraga et al (2017),^67^ Kenya	HIV-positive patients with alcohol use (selective)	Task-shifted CBT intervention delivered by paraprofessionals versus not clear, but most likely to mental health professionals	CBA modelled cohort: 13 440	Societal, 6 years, 2013, 3%, USD	Scale-up costs per site, training costs, treatment costs	HIV incidence, household productivity in monetary terms	Benefit–cost ratio is $1.13 per dollar invested	9, good
Gentilello et al (2005),^57^ USA	Trauma patients with alcohol use (selective)	Brief alcohol intervention and screening versus no brief intervention and screening	CBA model	Healthcare, 5 years, 2000, 3%, USD	Alcohol use screening costs, intervention costs, healthcare costs	Healthcare use in monetary terms	$3.81 for every dollar spent	8, fair
Goetzel et al (2014),^75^ USA	2458 workers at 121 small Colorado businesses (universal)	Comprehensive worksite health promotion programme versus doing nothing	ROI simulation model	Perspective is not clearly stated, most likely from employer and healthcare, 1 year, not reported, USD	Cumulative savings, programme cost and ROI cost	Ten modifiable health risks: high blood glucose, obesity, physical inactivity, poor nutrition/eating habits, tobacco use, high total cholesterol, high blood pressure, high alcohol consumption, high stress and depression	$2.03 for every dollar invested	5.5, poor
Havard et al (2012),^25^ Australia	Participants aged 14 years and older with AUDIT scores of 8 or more (selective)	Emergency department-based brief alcohol intervention with mailed personalised feedback for patients with problem drinking versus no further contact	CEA RCT	Hospital, 6 weeks, 2009, no discount, AUD	Treatment costs	Reduction in average weekly consumption in patients who exhibit risky drinking with alcohol-involved emergency department presentations	AUD$0.48 ($0.40) for every one standard drink reduction in average weekly consumption in patients who exhibit risky drinking with alcohol-involved emergency department presentations	6.5, fair
Henke et al (2011),^38^ USA	Full-time employees aged 18–64 years who completed a health assessment (selective)	Johnson & Johnson programme versus no or partial intervention	ROI observational study (*n* = 31 823)	Company employee, 6-year follow-up, 2009, no discounting, USD	Intervention cost, medical care cost-saving	Medical care cost-saving	ROI ratio $3.92 for every dollar spent or $1.88 in conservative estimates. Company employees benefited from meaningful reductions in rates of obesity, high blood pressure, high cholesterol, tobacco use, physical inactivity and poor nutrition	5.5, poor
Holm et al (2014),^51^ Denmark	16 years and older (universal)	Tax increase 30%, increased minimum legal drinking age, advertising bans, limited hours of alcohol retail sales, brief interventions by telephone, longer intervention offered in municipal prevention centres, do nothing	CUA model	Health sector, 100 years, 2009, 3%, Euro	Intervention cost, healthcare cost-saving	DALY	Tax increase, advertising bans, reduced retail opening hours, brief intervention is cost-saving. ICER of minimum legal drinking age is €5661 ($876) per DALY averted and ICER of longer intervention is €62 955 ($9739) per DALY averted	9.5, good
Holm et al (2014),^50^ Denmark	16 years and older (universal)	Tax increase 20% Tax increase 100% Tax decrease 20% Do nothing	CUA model	Health sector, 100 years, 2009, 3%, Euro	Intervention cost, healthcare cost-saving	DALY	Tax increase is a cost-saving option whereas reducing alcohol tax is less effective and more costly than current practice	9, good
Hunter et al (2017),^83^ Italy	Participants (over 18 years) who screen positively for hazardous drinking (indicated)	Face-to-face brief intervention (*n* = 416) versus facilitated access to website (*n* = 347)	CUA RCT (*n* = 763)	Health sector, 1 year, 2015/2016, no discount, Euros	Intervention cost, healthcare cost-saving	QALY	No significant differences in QALY between the two groups. Facilitated access to website is associated with lower cost than face-to-face brief intervention	8, fair
Kapoor et al (2009),^84^ USA	Adult men and women in primary care with unhealthy alcohol use (indicated)	%CDT testing both alone and combined with questionnaire	CUA Economic modelling –literature based Markov and decision	Societal, lifetime (100 years), 2006, 3%, USD	Screening, direct healthcare costs	QALY	Compared with questionnaire only, no screening and %CDT only were dominated, whereas questionnaire plus %CDT ICER was $15 500 ($19 334)/QALY	8, fair
Kouimtsidis et al (2017),^85^ UK	Adults (18–65 years) with alcohol consumption and intellectual disabilities (selective)	Extended brief interventions (*n* = 15) compared with usual care (*n* = 15)	CEA RCT (*n* = 30)	Not reported, 3 months, not reported, GBP	Therapist training, appointment, average cost of intervention	AUDIT score, RCQ score, CORE-LD score, QALY for both treatment groups	Extended brief intervention cost per patient is £430 ($667). No difference in EQ-5D scores in the two groups	6.5, fair
Kruger et al (2014),^35^ UK	Young students beginning studies at Sheffield University (selective)	Online health behaviour intervention (U@Uni) and U@Uni roll-out	CEA, CUA RCT and model (*n* = 1445)	UK Department of Health, 6 months for RCT and lifetime for model, 2012, 1.5%, GBP	U@Uni cost (full intervention development) and U@Uni cost (roll-out) costs	long-term costs, life-years and QALY	Additional U@Uni cost £326 ($526) for additional 0.0013 QALYs per person. Mean costs intervention:control £474.96:£148.69 ($766:$240). ICER for U@Uni of £243 926 ($393 616) for additional QALY gain. Long-term model: U@Uni ICER would be £22 844 ($36 863) per additional QALY gained	8, fair
Kunz et al (2004),^26^ USA	Clients at the emergency department with problem drinking (indicated)	Alcohol screening and brief intervention (SBI) (*n* = 90) compared with control (*n* = 104)	CEA RCT (*n* = 294)	Societal, 3 months, not reported, USD	Salaries for staff, equipment, patient incentives and overhead, screening cost, time spent, etc.	Reduction of AUDIT score, average weekly number of drinks, heavy episodic drinkers	Per case intervention cost $632 ($876); 2.45 audit score; 2.89 drinks reduced per week for SBI. Raw cost-effectiveness ratio is $218.70 ($303) per drinks reduced per week; $257.9 ($357) per audit score reduction; $61.11 ($85) per 1 percentage point reduction in the follow-up probability of heavy drinking	7, fair
Lai et al (2007),^77^ Estonia	General population and heavy alcohol drinkers (universal)	A combination of interventions: advertising ban, increase tax, brief advice, reduced access to retail outlet and roadside breath testing	CEA, CUA Modelled (all Estonian general adult population)	Societal, 10-year intervention, 100-year follow up for model, 2000, 3%, Euro	All patient-related costs, programme costs (intervention, training, media etc), tax revenue excluded	DALY	Individual intervention: 1000–3000 DALYs averted annually; combination: 7500 DALYs averted annually. Imposing taxes are most cost-effective intervention to reduce alcohol consumption and smoking: €49 ($195) and €14 ($56) per DALY averted. All interventions were more costly and more effective compared with doing nothing	9, good
Li et al (2017),^58^ USA	Participants with alcohol misconduct in military setting (indicated)	Brief alcohol intervention (Alcohol Misconduct Prevention Program (AMPP)) compared with control group	CEA, CBA Conservative modelling (*n* = 33 560 airmen)	USAF, not stated, 2013, not reported, USD	Programme cost/intervention costs	Number of alcohol-related incidents, cost-effectiveness ratio, cost–benefit ratio	$9869 ($10 896) for every alcohol-related incident avoided, the USAF saved $4.09 per dollar invested in the conservative model without health effects, and $6.17 per dollar invested when accounting health benefits. Sensitivity robust and favourable for the USAF	6.5, fair
Lock et al (2006),^86^ USA	Clients in primary healthcare with excessive alcohol use (indicated)	Nurse-led screening and brief intervention (*n* = 67) compared with standard management (*n* = 60)	CEA RCT (*n* = 127)	NHS and individual, 12 months, 2001/2002, USD	Resource used (GP cost, nurse cost, in-patient and out-patient care, emergency care), patient costs (travel, opportunity costs)	AUDIT score, average number of drinks per day, Drinking Problems Index,	Mean costs $291 ($403) *v*. $392 ($543) intervention versus control. Difference was non-significant, clinical outcome was non-significant	3.5, poor
Månsdotter et al (2007),^44^ Sweden	Victims of violence (selective)	Community mobilisation, responsible beverage service training and law enforcement	CUA pre–post observational, (*n* = 604)	Societal, 5 years, 1995, 3%, Euro	Administration, alcohol serving practices, community mobilisation, training and stricter alcohol law enforcement	QALY	Average cost of a violent crime €19 049 ($3186), which implies overall savings of €31.314 million ($5.24 million), cost-saving ratio was 1:39, 236 gained QALYs for society	6.5, fair
Miller et al (2007),^39^ USA	Employee (universal)	The PeerCare with drug testing	CBA retrospective ecological study	Employer, unclear, 1999, unclear, USD	Programme costs and drug testing costs	Injury related cost-saving	The peer-based programme costed the company $35 ($51) and testing cost another $35 ($51) per employee. The programme avoided an estimated $1850 ($2721) in employer injury costs per employee in 1999, corresponding to a benefit:cost ratio of 26:1	6.5, fair
Mundt et al (2006),^27^ USA	High-risk drinkers (indicated)	Screening and brief intervention in primary care versus control group	CBA RCT (*n* = 17 clinics and 64 physicians and internists)	Medical payer and societal, 48 months, USD	Clinical and hospital costs, intervention costs (staff training, screening for problem alcohol use, assessment of participants’ appropriateness for intervention, the intervention itself, follow-up and patient time and travel costs)	Benefits in monetary terms: reduced hospital and emergency department use, fewer criminal and legal events, and fewer motor vehicle incidents	Societal benefit:cost ratio was 39:1	4.5, poor
Nadkarni et al (2017),^22^ India	Adult males with harmful alcohol use (indicated)	Counselling for alcohol problems plus enhanced usual care (*n* = 188) versus enhanced usual care alone (*n* = 189)	CEA, CUA RCT (*n* = 377)	Health sector and societal, 1 year, 2015, no discount, USD	Heath service costs, time and travel costs, productivity loss costs	Remission Recovery QALY	The intervention was cost-saving	9, good
Navarro et al (2011),^59^ Australia	People who exhibit risky drinking (indicated)	Increase proportion of people undergoing screening and/or brief intervention	CEA model	Unclear (probably societal), 1 year, 2005/2006, no discount, AUD	Intervention costs	Reduction in patients who exhibit risky drinking	The most cost-effective outcome per additional patient reducing their drinking, relative to current practice, would be for GPs to screen all patients who exhibit risky drinking, with an ICER of AUD$197 ($184)	6.5, fair
Neighbors et al (2010),^68^ USA	Youth aged 18–19 years admitted to the emergency department with alcohol-related injuries (indicated)	Motivational interviewing-based intervention relative to brief advice to stop alcohol-related risk behaviours (standard care)	CEA, CUA model	Societal and provider, 6 months, 2008, no discount, USD	Intervention costs, fatal accident costs	Incidence of drink-driving, alcohol-related injuries, vehicular citations, alcohol problems, life-years, QALY	$362.04–$375.96 ($431–$448) per incidence of drink-driving avoided; $591.33–$614.07 ($705–$732) per alcohol-related injury incident avoided; $387.34–$402.23 ($462–$479) per traffic ticket avoided; $953.76–$990.43 ($1136–$1180) per alcohol problem avoided; $2414 ($2876) per QALY (men); $121 469 ($144 736) per QALY (women); $8795 ($10 480) per QALY (combined)	9.5, good
Paltzer et al (2019),^42^ USA	General Medicaid population (Wisconsin), 18–64 years (selective)	SBIRT (*n* = 7192) and usual care (*n* = 7664)	CEA pre–post study (*n* = 14 856) with modelling	No perspective reported, 24-months, 2018, no discounting reported, USD	Out-patient day costs, in-patient day costs, emergency department admission costs, SBIRT costs	Out-patient days, in-patient length of stay, in-patient and emergency department admissions	No ICER reported. No significant differences in out-patient/in-patient days, in-patient admissions and emergency department admissions. Cost offset from savings was –$781.97 (−$796). Cost of SBIRT was $51.05 ($52)	6, fair
Patra et al (2011),^60^ Canada	Canadian population, 19–21 years and older than 15 years (other interventions) (universal)	Taxation increases, lowering the BAC legal limit from 0.08% to 0.05%, zero BAC restriction for all drivers under the age of 21 years, increasing the minimum legal drinking age from 19 to 21 years, a Safer Bars intervention, privatisation and brief interventions	CEA model	No perspective reported, 1 year time horizon, 2002/2003, no discounting, CAD	Liquor license cost, drug enforcement costs, adult correction costs	Criminality indicators (drink-driving, homicide and other violent crimes, and other alcohol-related criminalities	No ICERs reported. All interventions except privatisation show positive outcomes and costs (dominance). Privatisation is dominated	5, poor
Pringle et al (2018),^43^ USA	Adult patients that presented to the emergency department with Medicaid coverage and identified by an honest broker (selective)	Usual emergency department visit with SBIRT and usual emergency department services	CBA, CEA Retrospective analysis (pre–post) study	No perspective reported, 1-year, 2016 (assumption, not reported), no discounting reported, USD	Billed charges from general and behavioural health data	Total healthcare costs, 30-day emergency department visits, 1-year emergency department visits, in-patient claims and behavioural health claims	No ICERs reported. Pre–post results for intervention hospital were 21% [−$2074 ($2200), *P* < 0.001] decrease in healthcare costs; 3.3% (*P* = 0.004) decrease in 1-year emergency department visits; and 4.1% (*P* < 0.001) decrease in in-patient claims; and 0.04% (*P* = 0.595) decrease in out-patient behavioural health claims	4.5, poor
Purshouse et al (2010),^46^ UK	English adult population (universal)	Alcohol pricing policies	CEA model	Healthcare, 10 years, 2006/2007, 3.5% (costs) and 1.5% (QALYs), GBP	Deaths, illnesses, admissions, healthcare costs	Net savings	Policies that increase the price of alcohol would reduce consumption, leading to reductions in mortality, disease prevalence and admissions, and savings in healthcare costs	6, fair
Purshouse et al (2013),^61^ UK	English population (universal)	Different SBI delivery mechanisms compared with no programme	CUA modelling (Sheffield)	Health sector, 30 years, 2007, 3.5%, GBP	Implementation and delivery costs, social costs incurred by drinkers	QALY	£6900 ($10 930) per QALY gained (SBI versus no programme); a consultation approach, delivered by a doctor compared with current practice will yield a £1175 ($1861) per QALY gained ICER	8.5, fair
Quanbeck et al (2010),^62^ USA	Routine healthcare visit, patients identified as problem drinkers from the WHO's ASSIST (indicated)	SBIRT and do nothing	CBA model	Employer, 4 years, 2008 (not reported, assumption), 3.5%, USD	Absenteeism, impaired presenteeism, implementation costs	Absenteeism and impaired presenteeism, costs	CBA ratio was 4.4:1. Implementation cost was $227 ($270) per employee. Absenteeism cost reduction was $175 ($209). Impaired presenteeism cost reduction was $823 ($981). Net present value owing to SBIRT was $771 ($919).	8.5, fair
Rehm et al (2011),^47^ Canada	Canadian population with alcohol use (selective)	Six alcohol policy interventions (pricing and taxation, lowering BAC level, zero BAC restriction, raising of minimum legal drinking age, safer bars, brief intervention and privatisation versus state monopoly on alcohol sale compared with baseline costs	CEA model	Policy implementation, 1 year, 2002, not reported, CAD	Alcohol-attributable costs: healthcare, criminal and productivity losses	Mortality, years of life lost, acute care hospital days	ICER not performed. Would result in savings from about 600–700 lives, 20 000–23 000 years of life lost and 83 000 acute care hospital days in Canada per year. Cost-savings from about CAD $900 million to $1 billion ($1.04 billion $1.16 billion) per year	7, fair
Schramm et al (1977),^37^ USA	Identified workers with alcoholism (indicated)	Employee health programme and do nothing	ROI Pre–post study	No perspective reported, 12 months, 1973, no discounting, USD	Programme costs (liaison, administration, medical, counselling and overhead costs)	Reduced absenteeism	ROI ratio was −2.20 for the first year but was expected to turn positive in the second year	7, fair
Schulz et al (2014),^36^ The Netherlands	General population, 18–65 years, with computer and internet access with basic internet literacy (selective)	Web-based computer-tailored lifestyle intervention [sequential condition (*n* = 552) or simultaneous condition (*n* = 517)] and minimal intervention (*n* = 664)	CEA, CUA RCT (*n* = 1733)	Societal, 2 years, 2013, 4% (costs) and 1.5% (effects), Euro	Website hosting costs, healthcare costs (medication, consultations, in-patient/out-patient care, hospital admissions and other care), productivity and respondent (patient and family) costs	Lifestyle factor score, EQ-5D-3L	Incremental costs were €183.76 ($246) sequential versus control, €868 ($1163) simultaneous versus control and −€684.24 (−$917) sequential versus simultaneous. Incremental lifestyle factor scores were 0.04 (sequential versus control), 0.08 (simultaneous versus control) and −0.04 (sequential versus simultaneous). CEA results were €4594 ($6155) (sequential versus control), €10 850 ($14 536) (simultaneous versus control) and €17 106 ($22 917) (sequential versus simultaneous). Incremental QALYs were −0.01 (sequential versus control), −0.03 (simultaneous versus control) and 0.02 (sequential versus simultaneous). CUA results were dominated (sequential versus control), dominated (simultaneous versus control) and dominated (sequential versus simultaneous)	8.5, fair
Shakeshaft et al (2002),^87^ Australia	Clients of a free community-based drug and alcohol counselling service excluding under the influence of another substance or requiring immediate detoxification unit referral (selective)	Brief intervention (*n* = 147) and CBT (*n* = 148) in an out-patient setting	CEA RCT (*n* = 295)	Agency, 6 months, 2002 (assumption, not reported), no discounting, AUD	Salaries, training in treatment delivery and resource materials	Effectiveness index (from weekly consumption, binge episodes, drinking intensity, alcohol-related problems and AUDIT scores), costs	No ICERs reported. No statistical difference in outcomes. Intention-to-treat analysis resulted in mean costs $32.84 ($36) (brief intervention) and $76.53 ($84) (CBT). Intention-to-treat effectiveness index scores were 11.12 (brief intervention) and 11.45 (CBT). Cost-effectiveness ratios were $2.95 ($3) (brief intervention) and $6.69 ($7) (CBT). On-treatment analysis results for brief intervention and CBT were $34.62 ($38) and $103.38 ($113) (mean costs); 12.35 and 12.68 (index); and 2.80 and 8.15 (CERs), respectively	5.5, poor
Shepard et al (2016),^33^ USA	Heavy drinkers (AUDIT score 8 or above) and identified significant other (indicated)	Incorporation of significant other into motivational intervention (SOMI) and brief individual motivational intervention	CBA, CEA, CUA RCT (*n* = 406)	Societal and health systems, 1 year, 2014, no discounting (base year unit prices were used), USD	Treatment costs and overhead, client travel costs	Percentage of hazardous drinking, QALY, cost	Health system perspective ICERs were $3623 ($3925) per hazardous drinker averted at 12 months and $32 200 ($34 891) per QALY gained. Health system benefit–cost ratio weas $4.73 per dollar invested. Societal perspective ICERs were $4403 ($4771) per hazardous drinker averted and $39 100 ($42 368) per QALY gained. Societal benefit–cost ratio was $3.90 per dollar invested.	9, good
Tanaree et al (2019),^23^ Thailand	Intervention participants from Songkhla province, Southern Thailand, aged 15 and older (selective)	Integrated Management of Alcohol Intervention Program in the Health Care System (i-MAP)	ROI Mixed methods (interviews and cross-sectional surveys)	Societal, 5 years, 2017, 3% discount rate, Thai Baht	Pre-implementation costs, implementation resources, hospital data, productivity, overhead costs	Psychosocial outcomes, health and non-health services utilisation	ROI 2:1	10, good
Tariq et al (2009),^63^ The Netherlands	General Dutch population (20–65 years) (universal)	Opportunistic SBI and current scenario (no SBI)	CUA, CEA model	Healthcare, 100-year time horizon, 2008, 1.5% (effects) and 4% (costs), Euro	Costs of opportunistic screening, costs of brief intervention, the costs of alcohol-related diseases and costs of diseases unrelated to alcohol in life-years	QALY, life-years, costs	€5400 ($7534)/QALY gained and €3600 ($5023)/life-year gained mean ICERs	8.5, fair
Thanh et al (2015),^88^ Canada	Women served by FASD Cross-Ministry Committee. Alcohol user specific (indicated)	Parent–Child Assistance Program (P-CAP) – home visit/harm reduction intervention preventing alcohol-exposed births and no intervention scenario	CBA, CEA Economic modelling, decision tree	Societal, 3-year time horizon, 2013, 5%, CAD	Lifetime costs per case of FASD, intervention costs	Prevented FASD, costs	ICER $97 000 ($86 808) per prevented FASD case. NMB $22 million ($19.69 million)	8.5, fair
Van den berg et al (2008),^89^ The Netherlands	General Dutch population (universal)	Current taxation scenario compared with planned tax increase and high tax scenario	CEA, CUA model	Healthcare, lifetime (100 years), 2003, 4% costs and 1.5%, Euro	Healthcare costs	LY, QALY	Dutch scenario (2.7 cent increase per bottle of beer): €3500 ($5398) per life-year gained €5100 ($7866) per QALY gained (highest tax – all alcohol) scenario: €3600 ($5553) per life-year gained €5300 ($8175) per QALY gained	8.5, fair
Watson et al (2015),^90^ UK	Problem drinkers admitted to hospital (indicated)	Healthy living-focused intervention (*n* = 43) based on principles of behaviour change counselling with involvement of a supportive concerned other, or ‘buddy’ (where one was available), and alcohol-focused intervention (*n* = 43) based on social behaviour and network therapy	CEA, CUA Economic evaluation alongside RCT feasibility study (*n* = 86)	No perspective reported, 12-month time horizon, 2012/2013, no discounting, GBP	Treatment and overhead, healthcare and social services, and policing and criminal justice system costs	AUDIT, ASSIST, Leeds Dependence Questionnaire, Social Satisfaction Questionnaire, Clinical Outcomes in Routine Evaluation; 12-item (short version) Working Alliance Inventory, EQ-5D, costs	No ICER reported. Trial was not powered to detect difference. No difference found in AUDIT scores. Mean costs of resource use decreased over 6 months. Slight improvement in EQ-5D scores	5.5, poor
Watson et al (2015),^91^ UK	Employees of a Local Authority Council that identify as ‘heavy drinkers’ (indicated)	Brief intervention (by occupational health nurse) (*n* = 26) and no brief intervention (*n* = 29)	CEA, CUA Economic evaluation alongside RCT pilot (*n* = 55)	Unclear perspective (occupational health services perspective), 6-month time horizon, 2006/2007, no discounting, GBP	Treatment for drinking problems, primary and secondary healthcare use, social care and information services, income and employment, productivity, work-related accidents, self-assessed alcohol-related problems at work and criminal justice service	AUDIT score change, number of drinking days per week, maximum number of units in one day, total weekly consumption, QALY, costs	No ICER reported. Significant effect observed in mean AUDIT scores over time, but no significant effect for groups. Difference in costs were £344.50 ($611) per person. QALYs fell in both groups (−0.002 for intervention and −0.010 for control), giving a net advantage of 0.008 QALYs for the intervention	3.5, poor
Wutzke et al (2001),^64^ Australia	General Australian population (universal)	Brief intervention with different strategies. Control (no training and no ongoing support), minimal support (5 min initial training with no further contact), maximal support (5 min initial training with telephone contact and personal visits every other week) and do nothing (individual)	CEA model	Health Department, lifetime time horizon, 1996, 3%, AUD	Marketing to primary health physicians, training and support, and counselling costs	LY, costs	AUD$645 ($808)/ life-year saved (control versus do nothing), AUD$510 ($639)/ life-year saved (no support versus control), AUD$581 ($728)/ life-year saved (no support versus do nothing), AUD$787 ($986)/ life-year saved (maximal support versus no support), AUD$653 ($819)/ life-year saved (maximal support versus do nothing)	8, fair
Zur and Zaric (2016),^65^ Canada	Canadian population (17 years and older) (universal)	Screening (using AUDIT and AUDIT-C thresholds) with brief intervention (SBI) compared with no SBI (individual)	CEA, CUA modelling (microsimulation)	Health payer, lifetime time horizon, 2013, 3%, CAD	Treatment and mortality	QALY, life-year, costs, ICERs	AUDIT (threshold score of 8) with brief intervention results were $10 678 ($9556)/life-year and $8145 ($7289)/QALY (men), $35 222 ($31 521)/life-year and $12 613 ($11 288)/QALY (women), $12 299 ($11 007)/life-year and $8729 ($7812)/QALY (combined). AUDIT thresholds lower than 8 were also cost-effective. AUDIT-C (threshold score of 7) with brief intervention results were $10 766 ($9635)/life-year and $8292 ($7421)/QALY (men), $36 130 ($32 334)/life-year and $13 665 ($12 229)/QALY (women), $11 808 ($10 567)/life-year and $8723 ($7806)/QALY (combined). AUDIT-C thresholds lower than 7 were also cost-effective	8.5, fair
Older adults								
Mundt et al (2005),^76^ USA	Older adults aged 65 and older who exhibit at-risk drinking (selective)	Brief intervention (*n* = 87) versus general health booklet control (*n* = 71)	CBA RCT (*n* = 158)	Medical payer and societal, 24 months, 1996, 5%, USD	Screening and intervention costs, patient-related costs	Economic benefits in monetary terms: health services utilisation and other social consequence	Reported intervention costs $236 ($362) treatment and $3 ($5) control. Societal costs $5241 ($8046) for treatment and $6289 ($9654) for control. No significant difference in benefits	6.5, fair

CEA, cost-effectiveness analysis; RCT, randomised controlled trial; GBP, Great British Pound; GP, general practitioner; PFBA, personalised feedback and brief advice; eBI, electronic brief intervention; CUA, cost–utility analysis; NHS, National Health Service; PSS, personal social services; QALY, quality-adjusted life-year; ICER, incremental cost-effectiveness ratio; WTP, willingness to pay; USD, US dollars; STD, sexually transmitted disease; CBA, cost–benefit analysis; SA, sensitivity analysis; AUDIT, Alcohol Use Disorders Identification Test; SF-12, 12-item Short-Form Survey; SBI, screening and brief intervention; SBIRT, screening, brief intervention and referral to treatment; AUD, Australian dollars; DALY, disability-adjusted life-years; WHO; World Health Organization; CER, cost-effectiveness ratio; AUDIT-C, Alcohol Use Disorders Identification Test; MIFB, motivational interviewing with feedback; EQ-5D, Euro-QoL five dimension; CBT, cognitive–behavioural therapy; ROI, return on investment; %CDT, carbohydrate-deficient transferrin; RCQ, Readiness to Change Questionnaire; CORE-LD, Clinical Outcomes in Routine Evaluation; USAF, United States Air Force; BAC, blood alcohol concentration; CAD, Canadian dollar; ASSIST, Alcohol, Smoking, and Substance Involvement Screening Test; EQ-5D-3L, European Quality of Life 5 Dimensions 3 Level Version; FASD, foetal alcohol spectrum disorder; NMB, net monetary benefit.

a. Dollar conversion with the EPPI-Centre Cost Converter.

b. Quality assessment with the Drummond ten-point checklist.

### Children, adolescents and young adults

#### Trial-based economic evaluations

Six economic evaluations were done alongside randomised controlled trials (RCTs) of preventive interventions targeting children, adolescents and young adults. One evaluation also incorporated economic modelling.^15^ Intervention settings varied from school-based to family-based to e-health. The time horizons of the trial-based economic evaluations ranged from 4 months to 5 years. All studies were set in high-income countries, with the majority of the studies located in the USA (*n* = 3). The perspectives that were adopted included societal (*n* = 2) and healthcare sector (*n* = 2).

One school-based intervention for adolescents and their parents/carers was found to be cost-saving in reducing alcohol use and binge drinking episodes compared with education as normal.^16^ Electronic brief intervention (eBI) and personalised feedback with brief advice (PFBA) were evaluated against screening only; with screening dominating eBI and PFBA not being cost-effective compared to screening.^17^ A web-based game with feedback on alcohol awareness targeting adolescents aged 15–19 years was more effective and more costly compared with care as usual, with an incremental cost-effectiveness ratio (ICER) of $83 per reduction of one glass of alcohol or $192 per reduction of binge drinking occasion per 30 days.^18^ A community mobilisation strategy to reduce youth substance use, delinquency, violence and other problem behaviours was cost-saving compared with a control community, with a benefit–cost ratio of $8.22 per dollar invested.^19^ A teenage prevention programme had a 90% probability of being cost-effective compared with attention control, using a willingness-to-pay threshold of $118 per dollar invested.^20^

#### Model-based economic evaluations

Two model-based economic evaluation studies were included for children, adolescents and young adults.^15,21^ Both studies used a societal perspective, with one study using a 5-year time horizon and the other using a lifetime time horizon. The two studies separately evaluated a family-based intervention, a parenting-only intervention and an intervention involving alcohol screening and counselling visits. The family-based intervention, which was about parenting skills with child involvement, produced a benefit–cost ratio of $9.60 per dollar invested, whereas the parenting-only intervention had a benefit of $5.85 per dollar invested when compared with minimal contact.^15^ The alcohol screening intervention plus the provision of counselling to youth identified at high risk of alcohol harms over 5 years would be cost-effective under the willingness-to-pay threshold of $978 047 per life-year saved compared with standard care, if programme efficacy was estimated at 5.6%.^21^

### Adults

#### Trial-based economic evaluations

The review identified 30 trial-based evaluations targeting adults. Twenty-one evaluations were conducted alongside RCTs, with the remainder conducted alongside non-RCT studies. Most studies were conducted in high-income countries such as the USA (*n* = 15), followed by the UK (*n* = 7), Australia (*n* = 2) and The Netherlands (*n* = 2). Only two studies were conducted in low- and middle-income countries, including India and Thailand.^22,23^ CEA (*n* = 11) was commonly used followed by combinations of multiple frameworks (*n* = 8), CBA (*n* = 4), CUA (*n* = 4) and ROIs (*n* = 3). Most studies adopted perspectives from societal (*n* = 5), healthcare sector (*n* = 5) or both (*n* = 5), with time horizons ranging from 6 weeks to 6 years.

Economic evaluations conducted alongside RCTs evaluated several interventions, including brief intervention or brief advice (*n* = 11), motivational interviewing and/or counselling (*n* = 5), and internet/computer-based interventions (*n* = 3). Evaluations of brief interventions reported ICERs of $0.40 to $303 per reduction in drinks per week^24–26^ and a benefit–cost ratio of $39 per dollar invested.^27^ The results of brief advice varied from being not cost-effective when compared with health and lifestyle leaflet,^28^ to having benefit–cost ratios of $5.6 (at 6 months)^29^ and $39 (at 12 months)^30^ per dollar invested compared with a control group. Motivational interviewing or counselling reported a range of CEA results, from being dominated (i.e. more costly and less effective) when compared with assessment only,^31^ to cost-saving when compared with enhanced usual care.^22^ Motivational interviewing was found to be dominant (i.e. less costly and more effective) compared with minimal intervention.^32^ Incorporating a patient's significant other into motivational interviewing (SOMI) had benefit–cost ratios of $4.23 (societal) and $5.13 (healthcare) per dollar invested when compared with motivational interviewing only.^33^ Web-based alcohol interventions reported ICERs ranging from being dominated when compared with minimal intervention, to $393 616 per QALY gained against measurement only.^34–36^

Economic evaluations done alongside non-RCT studies involved study designs such as pre–post, quasi-experimental and retrospective. Evaluated interventions included work-based interventions (*n* = 3); brief intervention (*n* = 2); screening, brief intervention and referral to treatment (SBIRT) (*n* = 2); a health promotion programme called the Integrated Management of Alcohol Intervention Program (i-MAP) and a community programme. The work-based interventions generated mixed ROI ratios ranging from no cost-savings^37^ to $3.92^38^ for every dollar spent, and a benefit:cost ratio of 26:1^39^ when compared with doing nothing. Different delivery methods for brief intervention were evaluated against usual care, with no impact on outcomes found.^40^ Another evaluation compared brief intervention (one to five sessions from 5 min to 1 h) against brief treatment (five to 12 1-h sessions intended for patients with higher risk factors) and reported that brief intervention was not better in terms of reducing the probability of using any alcohol, but was better in reducing the proportion using alcohol to intoxication, days of alcohol use and days of alcohol use to intoxication.^41^ SBIRT was found to be cost-saving compared with usual care,^42^ with a 21% reduction in healthcare costs and significant reductions in 1-year emergency department visits compared with usual emergency services use.^43^ The health promotion intervention i-MAP generated an ROI ratio of 2:1,^23^ and the community-based or multicomponent alcohol prevention programme was found to be cost-saving in terms of addressing violent crime.^44^

#### Model-based economic evaluations

There were 33 model-based economic evaluations, including an RCT study^35^ that modelled a longer time horizon and a pre–post study^42^ utilising Monte Carlo simulation. Most evaluations were conducted in high-income countries, including the USA (*n* = 8), Australia (*n* = 6), Canada (*n* = 4), the UK (*n* = 4), Denmark (*n* = 2), The Netherlands (*n* = 2), Estonia (*n* = 1) and Italy (*n* = 1). Low- and middle-income countries included Kenya (*n* = 1) and Thailand (*n* = 1). There was also one evaluation conducted in a European context and two studies that used a global context. Most studies involved CUA (*n* = 13), followed by CEA (*n* = 7), CBA (*n* = 3) and ROI (*n* = 1). Nine studies used a combination of different economic evaluation frameworks. The majority of studies adopted a health sector perspective (*n* = 17) and nine studies adopted a societal perspective. Almost half of the studies used a lifetime time horizon (*n* = 15).

The majority of the model-based interventions included upstream interventions such as policy and taxation (*n* = 15). Most CEAs of policy and taxation found that it generated cost-savings.^45–48^ Studies that adopted the CUA framework reported results that were cost-saving when compared with doing nothing.^49–51^ Brief intervention, SBI and SBIRT were the second-most modelled interventions (*n* = 17). Modelling indicated that brief intervention and SBI were cost-effective.^51–66^ Cognitive–behavioural therapy showed cost-savings,^67^ and motivational interviewing was cost-effective under a willingness-to-pay threshold of $50 000 per QALY.^68^

The models identified in this review were primarily multistage life table models, with or without a preceding decision tree. Individual-based models such as microsimulation and Monte Carlo simulation were also used, whereas simpler decision tree models were less common. Multistage life tables were used to estimate the incidence, prevalence, remission and mortality of alcohol-related diseases and injuries based on population life tables. These models can predict the demographic consequences derived from introducing new interventions (because of changes in the key parameters of incidence, prevalence remission or mortality).^66^ The changes in alcohol use affect the mortality and prevalence of alcohol-related diseases (e.g. liver failure), as well as the mortality and incidence of alcohol-related injuries (e.g. road accidents). These reductions then influence overall rates of mortality and disability in the population.^51^ The most commonly used multistage life table models included the model developed for the Adverse Childhood Experiences Prevention study,^66^ the Sheffield Alcohol Policy Model,^61^ the Chronic Disease Model^69^ and the model developed for the WHO-CHOICE study.^55^ Each multistage life table model applied potential impact fractions (PIFs) to estimate treatment effects. The PIF is an epidemiological measure of effect that calculates the proportional change in average disease incidence, prevalence or mortality after a change in the population distribution of a risk factor exposure.^70,71^

There was substantial heterogeneity between the different models on the number of health conditions attributable to alcohol use. The most comprehensive models covered up to 22 health conditions attributable to alcohol use.^61^ Non-communicable diseases (NCDs) were the most common conditions, followed by alcohol-related injuries. Alcohol dependence/alcohol use disorders were the only mental disorders included in existing models.

### Model-based economic evaluations of interventions to prevent multiple risk factors, including alcohol use

Four studies evaluated preventive interventions that included multiple risk factors, including alcohol use. Cadilhac et al^72^ modelled a hypothetical cohort to evaluate the cost–benefit of feasible reductions in six common risk factors over a lifetime (without decay). These risk factors include tobacco smoking, inadequate fruit and vegetable consumption, excessive alcohol use, high body mass index, physical inactivity and intimate partner violence. Cadilhac et al corrected the joint effects by using the joint population attributable risk fraction that was outlined in the 2003 Australian Burden of Disease and Injury study^73^ and by the World Health Organization.^74^ This formula is based on the assumption that health risks are independent.^74^ Results showed that reducing these risk factors saved 2334 million Australian dollars for the 2008 Australian adult population (or 4022 million in 2019 US dollars).

A pre–post study conducted in the USA showed that the Health Risk Management programme to reduce ten risk factors in workers (i.e. poor eating habits, physical inactivity, tobacco use, excessive alcohol use, high stress, depression symptoms, high blood pressure, high total cholesterol and high blood glucose) would produce a return of $2.03 per dollar invested within 1 year of follow-up.^75^ Growth curve modelling of a company health promotion and lifestyle programme was evaluated for the benefit of reducing rates of obesity, high blood pressure, high cholesterol, tobacco use, physical inactivity and poor nutrition over a 6-year time horizon. Despite having no effect on average alcohol consumption, the programme showed a return of $3.92 per dollar spent when all benefits were accounted for.^38^ A recent economic evaluation alongside a trial conducted by Kruger et al found that within the 6-month follow-up, a theory-based online health behaviour intervention implemented in university was not cost-effective in reducing unhealthy eating, physical inactivity, binge drinking and smoking.^35^ However, by extrapolating the efficacy of the intervention over a lifetime and rolling out the intervention to other universities, the intervention became cost-effective, with an ICER of £1545 ($2493) per QALY gained. This result is well below the UK willingness-to-pay threshold of £20 000 (around $28 653) per QALY gained.

### Older adults

Only one study was found for older adults with excessive alcohol use. Although this CBA reported lower healthcare and societal costs favouring the intervention group, it also showed no significant differences in costs between the intervention and control groups.^76^

### Quality assessment results.

There were 26% of conflicts registered out of a possible 2277 pairings from the 33 quality assessment sub-items. Post-assessment internal consistency was calculated and resulted in a KR20 coefficient of 0.76, which indicates acceptable internal consistency. Most studies (84%) were fair (*n* = 43) or good (*n* = 15) quality. Only two economic evaluations done alongside trials, Kuklinski et al^19^ and Tanaree et al,^23^ met all ten points of the quality checklist. Less than half of the studies lacked a clear description of the competing alternatives, relevant costs and consequences because of non-inclusion or non-reporting of capital costs. Most studies did not adequately present and discuss study results in terms of implementation, generalisability and future directions. Further details are presented in Table 2.

**Table 2:** Quality assessment of included economic evaluations

Author	1. Was a well-defined question posed in answerable form?	2. Was a comprehensive description of the competing alternatives given (i.e. can you tell who did what to whom, where and how often)?	3. Was the effectiveness of the programme or services established?	4. Were all the important and relevant costs and consequences for each alternative identified?	5. Were costs and consequences measured accurately in appropriate physical units (e.g. hours of nursing time, number of physician visits, lost workdays, gained life-years)?	6. Were the cost and consequences valued credibly?	7. Were costs and consequences adjusted for differential timing?	8. Was an incremental analysis of costs and consequences of alternatives performed?	9. Was allowance made for uncertainty in the estimates of costs and consequences?	10. Did the presentation and discussion of study results include all issues of concern to users?	Yes	Cannot tell	No	Quality score	Judgement
Agus et al (2019),^16^ UK	Yes	Yes	Yes	No	Yes	Yes	Yes	Yes	Yes	Cannot tell	8	1	1	8.5	Fair
Angus et al (2014),^52^ Italy	Yes	Yes	Yes	No	Yes	Yes	Yes	Yes	Yes	Yes	9	0	1	9	Good
Angus et al (2017),^53^ Europe	Yes	Cannot tell	Yes	No	Yes	Yes	Yes	Yes	Yes	Yes	8	1	1	8.5	Fair
Babor et al (2006),^40^ USA	No	Yes	Yes	Cannot tell	Yes	Yes	Yes	Yes	Yes	Yes	8	1	1	8.5	Fair
Barbosa et al (2015),^54^ USA	Yes	No	Yes	Yes	Yes	Yes	Yes	Yes	Yes	Yes	9	0	1	9	Good
Barbosa et al (2017),^41^ USA	No	No	Yes	No	Yes	Yes	Yes	Yes	Yes	Yes	7	0	3	7	Fair
Barrett et al (2006),^24^ UK	Yes	No	Yes	No	Yes	Yes	Yes	Yes	Yes	Cannot tell	7	1	2	7.5	Fair
Blankers et al (2012),^34^ The Netherlands	Yes	No	Yes	Cannot tell	Yes	Yes	Yes	Yes	Yes	Cannot tell	7	2	1	8	Fair
Brennan et al (2014),^48^ UK	Yes	No	Yes	No	Yes	Yes	No	No	Yes	Cannot tell	5	1	4	5.5	Poor
Byrnes et al (2010),^82^ Australia	Yes	Yes	Yes	Cannot tell	Yes	Yes	Yes	Cannot tell	Yes	Cannot tell	7	3	0	8.5	Fair
Cadilhac et al (2011),^72^ Australia	No	Yes	Yes	No	Yes	Yes	Yes	No	Yes	Cannot tell	6	1	3	6.5	Fair
Chisholm et al (2004),^56^ global	Yes	Yes	Yes	Cannot tell	Yes	Yes	Yes	Yes	Yes	Cannot tell	8	2	0	9	Good
Chisholm et al (2018),^55^ global	Cannot tell	Yes	No	Yes	Yes	Yes	No	Yes	No	Cannot tell	5	2	3	6	Fair
Cobiac et al (2009),^66^ Australia	Yes	Yes	Cannot tell	Cannot tell	Yes	Yes	No	Yes	No	Cannot tell	5	3	2	6.5	Fair
Coulton et al (2017),^32^ UK	Yes	No	Yes	Yes	Yes	Cannot tell	Yes	Yes	No	Yes	7	1	2	7.5	Fair
Cowell et al (2012),^31^ USA	Yes	No	Yes	Yes	Yes	Yes	Yes	Yes	Yes	Cannot tell	8	1	1	8.5	Fair
Cowell et al (2018),^78^ USA	No	Yes	Yes	Yes	Yes	Yes	Yes	Yes	Yes	Yes	9	0	1	9	Good
Crawford et al (2015),^28^ UK	Yes	No	Yes	No	Yes	Yes	Yes	No	Yes	No	6	0	4	6	Fair
Deluca et al (2021),^17^ UK	Yes	Yes	Yes	Yes	Yes	Yes	Yes	Yes	No	Cannot tell	8	1	1	8.5	Fair
Ditsuwan et al (2013),^45^ Thailand	Yes	Yes	Yes	No	Yes	Yes	Yes	Yes	Yes	Yes	9	0	1	9	Good
Doran et al (2013),^49^ Australia	Yes	Yes	Yes	No	Yes	Yes	Yes	No	No	Cannot tell	6	1	3	6.5	Fair
Downs and Klein (1995),^21^ USA	Yes	Yes	Yes	No	No	No	No	Yes	Yes	No	5	0	5	5	Poor
Drost et al (2016),^18^ The Netherlands	Yes	Yes	Yes	No	Yes	Yes	Yes	Yes	Yes	Cannot tell	8	1	1	8.5	Fair
Fleming et al (2000),^29^ USA	Yes	No	Yes	Yes	Yes	Yes	Yes	Yes	Yes	Cannot tell	8	1	1	8.5	Fair
Fleming et al (2002),^30^ USA	Yes	No	Yes	Yes	Yes	Yes	Yes	Yes	No	Cannot tell	7	1	2	7.5	Fair
Galárraga et al (2017),^67^ Africa	Yes	Cannot tell	Yes	Yes	Yes	Yes	Yes	Yes	Yes	Cannot tell	8	2	0	9	Good
Gentilello et al (2005),^57^ USA	Yes	Yes	Yes	Cannot tell	Yes	Yes	Yes	No	Yes	Cannot tell	7	2	1	8	Fair
Goetzel et al (2014),^75^ USA	Cannot tell	Cannot tell	Yes	Cannot tell	Yes	Cannot tell	Yes	No	No	Cannot tell	3	5	2	5.5	Poor
Havard et al (2012),^25^ Australia	Yes	No	Yes	No	Yes	Yes	Yes	Yes	No	Cannot tell	6	1	3	6.5	Fair
Henke et al (2011),^38^ USA	No	No	Yes	Cannot tell	Yes	Yes	No	Yes	Cannot tell	Cannot tell	4	3	3	5.5	Poor
Holm et al (2014),^51^ Denmark	Yes	Yes	Yes	Cannot tell	Yes	Yes	Yes	Yes	Yes	Yes	9	1	0	9.5	Good
Holm et al (2014),^50^ Denmark	Yes	Yes	Yes	Cannot tell	Yes	Yes	Yes	Yes	Yes	Cannot tell	8	2	0	9	Good
Hunter et al (2017),^83^ Italy	Yes	No	Yes	Yes	Yes	Cannot tell	Yes	Yes	Yes	Cannot tell	7	2	1	8	Fair
Ingels et al (2013),^20^ USA	Yes	Yes	Yes	Yes	Yes	Yes	No	Yes	Yes	No	8	0	2	8	Fair
Kapoor et al (2009),^84^ USA	Yes	Cannot tell	Yes	No	Yes	Yes	Yes	Yes	Yes	Cannot tell	7	2	1	8	Fair
Kouimtsidis et al (2017),^85^ UK	Cannot tell	Yes	Yes	No	Yes	Yes	Yes	No	No	Yes	6	1	3	6.5	Fair
Kruger et al (2014),^35^ UK	Yes	No	Yes	Cannot tell	Yes	Yes	Yes	Yes	Yes	Cannot tell	7	2	1	8	Fair
Kuklinski et al (2015),^19^ USA	Yes	Yes	Yes	Yes	Yes	Yes	Yes	Yes	Yes	Yes	10	0	0	10	Good
Kunz et al (2004),^26^ USA	Yes	No	Yes	Cannot tell	Yes	Cannot tell	Yes	Yes	No	Yes	6	2	2	7	Fair
Lai et al (2007),^77^ Estonia	Yes	Cannot tell	Yes	Yes	Yes	Yes	Yes	Yes	Yes	Cannot tell	8	2	0	9	Good
Li et al (2017),^58^ USA	Yes	Yes	Yes	No	Yes	No	No	Yes	Yes	Cannot tell	6	1	3	6.5	Fair
Lock et al (2006),^86^ USA	Yes	No	Yes	No	Cannot tell	No	Yes	No	No	No	3	1	6	3.5	Poor
Månsdotter et al (2007),^44^ Sweden	Yes	No	Yes	No	Yes	Yes	Yes	No	Yes	Cannot tell	6	1	3	6.5	Fair
Miller et al (2007),^39^ USA	Cannot tell	No	Yes	No	Yes	Yes	Yes	Yes	No	Yes	6	1	3	6.5	Fair
Mundt et al (2005),^76^ USA	Yes	Cannot tell	Yes	Yes	Yes	Yes	Yes	No	No	No	6	1	3	6.5	Fair
Mundt et al (2006),^27^ USA	Yes	Cannot tell	Yes	No	No	Yes	No	Yes	No	No	4	1	5	4.5	Poor
Nadkarni et al (2017),^22^ India	Yes	Yes	Yes	No	Yes	Yes	Yes	Yes	Yes	Yes	9	0	1	9	Good
Navarro et al (2011),^59^ Australia	No	No	Yes	Cannot tell	Yes	Yes	Yes	Yes	No	Yes	6	1	3	6.5	Fair
Neighbors et al (2010),^68^ USA	Yes	Cannot tell	Yes	Yes	Yes	Yes	Yes	Yes	Yes	Yes	9	1	0	9.5	Good
Paltzer et al (2019),^42^ USA	No	Yes	Yes	No	Yes	Yes	No	No	Yes	Yes	6	0	4	6	Fair
Patra et al (2011),^60^ Canada	No	No	Yes	No	Yes	Yes	Yes	No	Yes	No	5	0	5	5	Poor
Pringle et al (2018),^43^ USA	No	Yes	Yes	No	Yes	No	No	Yes	No	Cannot tell	4	1	5	4.5	Poor
Purshouse et al (2010),^46^ UK	Yes	No	Yes	No	Yes	Yes	No	No	Yes	Yes	6	0	4	6	Fair
Purshouse et al (2013),^61^ UK	Yes	Cannot tell	Yes	N	Yes	Yes	Yes	Yes	Yes	Yes	8	1	1	8.5	Fair
Quanbeck et al (2010),^62^ USA	Yes	Yes	Yes	Cannot tell	Yes	Yes	N	Yes	Yes	Yes	8	1	1	8.5	Fair
Rehm et al (2011),^47^ Canada	Yes	Cannot tell	Yes	No	Yes	Yes	Yes	No	Yes	Cannot tell	6	2	2	7	Fair
Schramm et al (1977),^37^ USA	No	Yes	Yes	Yes	Yes	Yes	Yes	Yes	No	No	7	0	3	7	Fair
Schulz et al (2014),^36^ The Netherlands	Yes	No	Yes	Yes	Yes	Yes	Yes	Yes	Yes	Cannot tell	8	1	1	8.5	Fair
Shakeshaft et al (2002),^87^ Australia	Yes	Yes	Yes	No	Cannot tell	No	Yes	No	No	Yes	5	1	4	5.5	Poor
Shepard et al (2016),^33^ USA	Yes	No	Yes	Yes	Yes	Yes	Yes	Yes	Yes	Yes	9	0	1	9	Good
Spoth et al (2002),^15^ USA	Yes	No	Yes	Yes	Yes	Yes	Yes	Yes	Yes	Yes	9	0	1	9	Good
Tanaree et al (2019),^23^ Thailand	Yes	Yes	Yes	Yes	Yes	Yes	Yes	Yes	Yes	Yes	10	0	0	10	Good
Tariq et al (2009),^63^ The Netherlands	Yes	Yes	Yes	Cannot tell	No	Yes	Yes	Yes	Yes	Yes	8	1	1	8.5	Fair
Thanh et al (2015),^88^ Canada	Yes	Yes	Yes	Cannot tell	Yes	Yes	Yes	Yes	Yes	No	8	1	1	8.5	Fair
van den Berg et al (2008),^89^ The Netherlands	Yes	Yes	Yes	No	Yes	Yes	Yes	Yes	Yes	Cannot tell	8	1	1	8.5	Fair
Watson et al (2015),^91^ UK	No	No	Yes	Yes	Yes	No	Yes	No	Yes	Cannot tell	5	1	4	5.5	Poor
Watson et al (2015),^91^ UK	No	Cannot tell	Yes	No	Yes	No	Yes	No	No	No	3	1	6	3.5	Poor
Wutzke et al (2001),^64^ Australia	Yes	No	Yes	No	Yes	Yes	Yes	Yes	Yes	Yes	8	0	2	8	Fair
Zur and Zaric al (2016),^65^ Canada	Yes	Cannot tell	Yes	No	Yes	Yes	Yes	Yes	Yes	Yes	8	1	1	8.5	Fair

### Colour grading of cost-effectiveness results

**Fig. 2:**
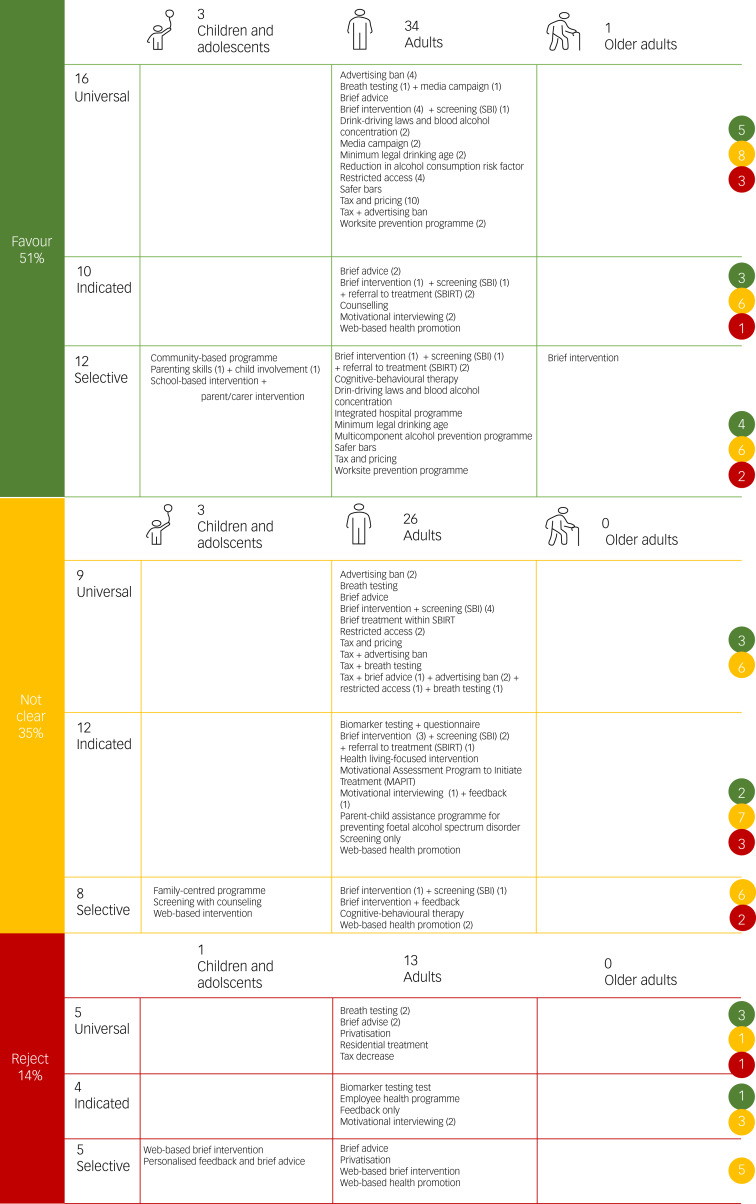
Cost-effectiveness summary of interventions. SBI, screening and brief intervention; SBIRT, screening, brief intervention and referral to treatment.

Figure 2 presents a summary of the classification for different interventions graded based on their results and grouped as either likely to be rejected, favoured or unclear from a decision-making perspective. Half of the interventions were found to be cost-saving for the prevention of alcohol use and 84% of studies were rated as either fair (53%) or good (32%) quality. Most interventions were delivered to adults or the general population, except for four interventions targeting children and their parents and one intervention for older adults. Specifically, universal prevention strategies restricting access to alcohol through taxation or advertising bans and selective/indicated prevention through screening with or without brief intervention accounted for most of the studies.

Another 35% of interventions were categorised as ‘unclear’ because they produced improved health outcomes at a higher cost. Two-thirds of economic evaluations were fair quality, followed by studies rated as good (17%) or poor (17%) quality. Most interventions restricted exposure to alcohol through taxation with or without advertising bans for general populations or selective/indicated prevention through screening with or without brief intervention for targeting adults.

A total of 14% of interventions from the economic evaluations were categorised as ‘reject’ (i.e. less effective and more costly). Around a quarter of these studies were good quality, and two-thirds were fair quality.

Several interventions show cost-effectiveness results with a high degree of uncertainty, meaning that they comprise cost-effectiveness evidence that simultaneously indicate ‘favour’, ‘unclear’ and ‘reject’ decisions. These mixed results were affected by the variance around the choice of study elements. This suggests that a particular intervention may be acceptable or not appropriate in certain situations or contexts. For example, in universal interventions, both breath testing and brief advice had varying cost-effectiveness results. The ‘favour’ judgement for breath testing was from a modelling study showing cost-savings when compared with doing nothing.^45^ However, two other studies modelled both breath testing and brief advice against different comparators, with one comparing both with current situation^77^ and the other using taxation as the comparator.^56^ In the first study, both breath testing and brief advice had more costs and less DALYs compared with current situation in the Estonian population.^77^ In the second, a global regional modelling study, brief advice had cost-effectiveness results ranging from ‘favour’ in some regions (because it was dominant) to ‘reject’ in other regions (because it was dominated by taxation).^56^ The study reported that generally, both interventions incurred higher costs than taxation. In terms of DALYs, regions with higher levels of heavy alcohol use lean toward taxation being more effective, whereas breath testing and brief advice are more effective in regions with less prevalence of heavy alcohol use.^56^

In terms of ‘indicated’ interventions for adults, motivational interviewing showed cost-effectiveness judgements ranging from ‘favour’ to ‘reject’. SOMI reported ‘favour’ judgement from a positive cost–benefit ratio in both healthcare and societal perspectives when compared with standard motivational interviewing. Standard motivational interviewing reported ‘unclear’ cost-effectiveness results compared with standard care.^68^ Two motivational interviewing studies reported ‘reject’ results because it was dominated by motivational interviewing with feedback^31^ and by the intervention Motivational Assessment Program to Initiate Treatment (MAPIT).^78^ Motivational interviewing studies that used societal perspectives were judged ‘favour’ or ‘unclear’, whereas ‘reject’ cost-effectiveness results were found from narrower provider and probationary system perspectives.

In ‘selective’ interventions, web-based health promotion had both ‘unclear’ and ‘reject’ judgements. A study reported cost-effectiveness results for web-based health promotion judged as ‘unclear’ because of higher costs and higher utilities for the intervention compared with doing nothing, in a population of university students. The analysis was over 6-month and lifetime time horizons, and only used intervention and rollout expenses for costs.^35^ In another study, web-based intervention was compared with minimal intervention. Reported results were mixed with ‘unclear’ judgement for CEA (lifestyle factor score improvement outcome) and ‘reject’ judgement for CUA. This evaluation was from a societal perspective and over a 2-year time horizon for the people with computer and internet access with basic internet literacy.^36^

## Discussion

The current review provides an update on the cost-effectiveness evidence for the prevention of alcohol use across the lifespan. The number of studies included in this review is nearly two and a half times more than those included in a previous review.^6^ Most of the cost-effectiveness evidence has been evaluated for interventions targeting adults. There were limited economic evaluations of interventions targeting children, adolescents and older adults. Furthermore, most studies were conducted in high-income countries, particularly using trial-based economic evaluations. Less cost-effectiveness research has been undertaken in low- and middle-income countries. Half of the evidence estimated that preventive interventions for alcohol use were cost-saving. The interventions frequently found to be cost-saving were ‘universal’ prevention, consisting largely of increasing the price of alcohol via taxation or reducing exposure to alcohol via advertising bans. Most of these interventions were compared against doing nothing or having no policy in place. Selective/indicated prevention, such as screening with or without brief intervention, was also found to be cost-saving when compared with doing nothing. It is also encouraging that school-based interventions, with and without interventions for parents (selective prevention), were found to be cost-saving, albeit in a limited number of studies. However, it is important to note that it is difficult to determine which intervention is the optimal choice, given that little evidence was established to compare different interventions within a single-study context. In terms of study quality, most studies included in this review had fair to good quality.

The results of this review provide important economic evaluation evidence to support the implementation of alcohol prevention interventions at a population level. However, it should be noted that although economic evaluation offers a useful format (with one concise indicator) for decision-making, it is not a perfect instrument. In particular, results across different economic evaluations are not comparable because of the variations in methodology, such as how utility scores for calculating QALYs were measured (differences in outcome measurement tool used) or the context in which the intervention was conducted. Other implementation considerations, such as equity, feasibility, sustainability and acceptability, may not be adequately addressed by economic evaluations. Therefore, even if there is clear evidence of effectiveness or cost-effectiveness, it does not necessarily guarantee intervention uptake.

The conflicting cost-effectiveness findings observed across several interventions were the result of substantive variations in study design. These variations limit the ability to conduct any prospective meta-analysis, highlighting a limitation of economic evaluations. Chisholm et al effectively demonstrated this issue where differences in study design or data inputs from different countries resulted in varying cost-effectiveness estimates.^56^ In their evaluation, alcohol taxation was-cost saving in the USA and European countries; however, it was found to be more effective and more costly in African and Asian countries, with ICERs under a willingness-to-pay threshold of $50 000 per DALY.^56^ Therefore, it is important that policy decisions be aided by adequate, context-specific research to determine which interventions can be considered value for money.

The paucity of cost-effectiveness studies on alcohol prevention among children and adolescents is in stark contrast to the literature on the cost-effectiveness of mental health promotion and prevention, where most of the existing research has focused on children, adolescents and youth.^79^ Prevention of alcohol use in adolescents is important given that early use of alcohol predicts frequent drinking, leading to future alcohol-related harms.^80^ Furthermore, the frequency of adolescent drinking is also predictive of substance use problems in adulthood. Further research is urgently needed to establish the value-for-money credentials of interventions to prevent or delay alcohol use in this age group.

Trial-based economic evaluations primarily evaluated indicated prevention interventions, whereas model-based economic evaluations primarily evaluated universal prevention interventions. This is sensible because universal preventive interventions are expected to have broad effects that may take years to be realised and are difficult to properly evaluate in a trial. Furthermore, model-based economic evaluations can estimate the long-term effects of alcohol use, including its effects on NCDs. Ideally, it is important to capture the full breadth of long-term effects produced by alcohol prevention interventions over the life course. However, the effect of prevention interventions over the long term becomes more uncertain, as extrapolating the longer-term effects of an intervention typically necessitates the use of assumptions that are not based on empirical evidence. Furthermore, the effects of intervention have been found to attenuate over time in long-term follow-up studies.

This review also included economic evaluations evaluating multiple risk factors, including alcohol use. However, only four studies were found that focused on combined alcohol use and risk factors for physical health conditions (e.g. obesity or NCDs). There is currently no evidence on the cost-effectiveness of preventive interventions for alcohol use and risk factors for mental health conditions. Given the high prevalence of comorbidity between alcohol use disorder, other drug use disorders and mental disorders,^81^ further research should explore the impact of interventions on risk factors for both physical and mental health conditions.

This review has several limitations. Only peer-reviewed articles published in the English language were included, which may have contributed to the lack of studies conducted in low- and middle-income countries. It is also common for economic evaluations, especially ROI studies, to be published in grey literature rather than in the academic literature, potentially limiting the studies identified. In addition, the involvement of multiple reviewers in screening and extraction may have resulted in inconsistencies. Meta-analysis was also not possible given the high level of methodological heterogeneity in the populations, interventions, comparators and outcomes, as well as economic evaluation frameworks across included studies. Furthermore, the majority of cost-effectiveness evidence supported the prevention of alcohol use, raising concerns of publication bias. Alternatively, a strength of this review is the use of a dominance ranking framework to summarise and provide recommendations for policy and practice.^79^

In conclusion, this study found that prevention interventions for alcohol use are promising and likely provide good value for money. These findings will be of value to policy makers and other stakeholders interested in preventing alcohol use and/or excessive alcohol use. Nevertheless, policy decisions should still be aided by adequate, context-specific research on possible prevention interventions, to determine whether such interventions would be value for money. Future economic analyses are needed for low- and middle-income countries, as well as for children, adolescents and older adults. Moreover, research on cost-effectiveness with longer follow-up is also required, as it is uncertain whether the modelled longer-term effects of interventions will, in fact, be realised.

## Data Availability

The authors confirm that the data supporting the findings of this study are available within the article and its supplementary materials.

## Supplementary material

For supplementary material accompanying this paper visit http://doi.org/10.1192/bjo.2023.81. click here to view supplementary material
