# Supplementary material for: Interventions to prevent alcohol use: systematic review of economic evaluations
Source: BJPsych Open. 2023 Jun 27;9(4):e117. doi: 10.1192/bjo.2023.81 (PMC10305038; doi:10.1192/bjo.2023.81)
Supplement: Supplementary file 1 [file S2056472423000819sup001.docx]

Appendix 1: Search strategy (Embase)

| No. | Query | Results  (01/08/2019) | Results  (05/05/2021) |
| --- | --- | --- | --- |
| #1 | 'substance use'/exp | 468,870 | 528,583 |
| #2 | 'alcohol abuse'/exp | 36,642 | 42,102 |
| #3 | 'drug abuse'/exp | 109,851 | 124,284 |
| #4 | 'street drug'/de | 3,587 | 3,700 |
| #5 | ((alcohol OR drug* OR substance* OR 'ill* drug*' OR 'banned substance*' OR cannabi* OR marijuana OR cocaine OR heroin OR meth* OR 'street drug*') NEAR/2 (use OR misuse OR abuse OR intake OR consum*)):ti | 85,827 | 96,795 |
| #6 | ((alcohol OR drug* OR substance* OR 'ill* drug*' OR 'banned substance*' OR cannabi* OR marijuana OR cocaine OR heroin OR meth* OR 'street drug*') NEAR/2 (use OR misuse OR abuse OR intake OR consum*)):ab | 333,755 | 382,032 |
| #7 | #1 OR #2 OR #3 OR #4 OR #5 OR #6 | 775,862 | 876,252 |
| #8 | prevent*:ti OR promot*:ti OR treat*:ti OR manag*:ti | 2,783,298 | 1,207,789 |
| #9 | prevent*:ab OR promot*:ab OR treat*:ab OR manag*:ab | 8,755,720 | 4,386,303 |
| #10 | #8 OR #9 | 9,844,974 | 4,922,369 |
| #11 | #7 AND #10 | 307,913 | 182,321 |
| #12 | 'cost benefit analysis'/de | 81,166 | 86,660 |
| #13 | 'cost benefit analysis':ti | 1,693 | 1,791 |
| #14 | 'cost benefit analysis':ab | 3,589 | 3,928 |
| #15 | 'economic evaluation'/exp | 290,692 | 317,786 |
| #16 | 'economic evaluation*':ti | 6,400 | 7,297 |
| #17 | 'economic evaluation*':ab | 11,654 | 13,757 |
| #18 | 'cost analys?s':ti | 3,907 | 4,463 |
| #19 | 'cost analys?s':ab | 7,668 | 8,831 |
| #20 | 'return on investment':ti | 404 | 467 |
| #21 | 'return on investment':ab | 1,942 | 2,354 |
| #22 | 'return to investment':ti | 1 | 1 |
| #23 | 'return to investment':ab | 8 | 9 |
| #24 | 'cost effectiveness analysis'/de | 142,752 | 158,570 |
| #25 | 'cost effectiveness analysis':ti | 6,786 | 7,986 |
| #26 | 'cost effectiveness analysis':ab | 8,653 | 10,014 |
| #27 | 'cost utility analysis'/de | 9,006 | 10,297 |
| #28 | 'cost utility analysis':ti | 1,491 | 1,714 |
| #29 | 'cost utility analysis':ab | 2,476 | 2,858 |
| #30 | #12 OR #13 OR #14 OR #15 OR #16 OR #17 OR #18 OR #19 OR #20 OR #21 OR #22 OR #23 OR #24 OR #25 OR #26 OR #27 OR #28 OR #29 | 298,098 | 326,149 |
| #31 | #11 AND #30 | 5,574 | 3,739 |
| #32 | #11 AND #30 AND [humans]/lim AND [english]/lim AND [embase]/lim | 4,266 | 2,880 |
| #33 | #11 AND #30 AND ([article]/lim OR [article in press]/lim OR [erratum]/lim) AND [humans]/lim AND [english]/lim AND [embase]/lim | 2,068 | 1,321 |
|  |  | 2,187 | |

Note: The highlighted term ‘treat*’ was not included in the 05/05/2021 to reflect the preventive focus of this review.

Search strategy (Medline)

| # | Query | Results  (01/08/2019) | Results  (05/05/2021) |
| --- | --- | --- | --- |
| S1 | (MH "Marijuana Use") OR (MH "Marijuana Abuse") OR (MH "Inhalant Abuse") OR (MH "Cocaine-Related Disorders") OR (MH "Amphetamine-Related Disorders") OR (MH "Alcoholism") OR (MH "Binge Drinking") OR (MH "Alcoholic Intoxication") OR (MH "Substance Abuse, Intravenous") OR (MH "Substance Abuse, Oral") OR (MH "Tobacco Use Disorder") OR (MH "Phencyclidine Abuse") OR (MH "Drug Overdose") | 132,849 | 141,868 |
| S2 | (MH "Alcohol Drinking+") | 65,396 | 71,123 |
| S3 | (MH "Crack Cocaine") | 1,378 | 1,441 |
| S4 | TI (alcohol OR drug* OR substance* OR "ill* drug*" OR "banned substance*" OR cannabi* OR marijuana OR cocaine OR heroin OR meth* OR "street drug*") N2 (use or misuse or abuse or intake or consum*) | 75,784 | 87,598 |
| S5 | AB (alcohol OR drug* OR substance* OR "ill* drug*" OR "banned substance*" OR cannabi* OR marijuana OR cocaine OR heroin OR meth* OR "street drug*") N2 (use or misuse or abuse or intake or consum*) | 293,536 | 332,602 |
| S6 | S1 OR S2 OR S3 OR S4 OR S5 | 428,580 | 476,835 |
| S7 | TI prevent* or promot* or treat* or manag* | 7,418,339 | 3,149,512 |
| S8 | AB prevent* or promot* or treat* or manag* | 7,817,921 | 3,940,864 |
| S9 | S7 OR S8 | 7,927,888 | 4,092,459 |
| S10 | S6 AND S9 | 176,339 | 99,231 |
| S11 | (MH "Cost-Benefit Analysis") | 76,614 | 84,003 |
| S12 | TI "Cost Benefit Analys?s" | 1,323 | 1,444 |
| S13 | AB "Cost Benefit Analys?s" | 3,213 | 3,589 |
| S14 | TI "economic evaluation*" | 4,747 | 5,339 |
| S15 | AB "economic evaluation*" | 8,199 | 9,588 |
| S16 | (MH "Costs and Cost Analysis") | 47,259 | 49,429 |
| S17 | TI "Cost Analys?s" | 2,620 | 2,980 |
| S18 | AB "Cost Analys?s" | 4,771 | 5,473 |
| S19 | TI "return on investment" | 331 | 402 |
| S20 | AB "return on investment" | 1,482 | 1,801 |
| S21 | TI "return to investment" | 1 | 1 |
| S22 | AB "return to investment" | 5 | 5 |
| S23 | TI "cost effectiveness analys?s" | 4,666 | 5,421 |
| S24 | AB "cost effectiveness analys?s" | 7,607 | 8,718 |
| S25 | TI "cost utility analys?s" | 982 | 1,128 |
| S26 | AB "cost utility analys?s" | 2,152 | 2,549 |
| S27 | S11 OR S12 OR S13 OR S14 OR S15 OR S16 OR S17 OR S18 OR S19 OR S20 OR S21 OR S22 OR S23 OR S24 OR S25 OR S26 | 134,861 | 146,378 |
| S28 | S10 AND S27 | 2,236 | 1,378 |
| S29 | S10 AND S27 (Limiters – English Language; Human; Publication Type: Introductory Journal Article, Journal Article Search modes - Boolean/Phrase) | 1,888 | 1,184 |
|  |  | 2,017 | |

Note: The highlighted term ‘treat*’ was not included in the 05/05/2021 to reflect the preventive focus of this review.

Search strategy (Cinahl)

| # | Query | Results  (01/08/2019) | Results  (05/05/2021) |
| --- | --- | --- | --- |
| S1 | (MH "Substance Abuse+") | 60,314 | 70,418 |
| S2 | (MH "Substance Abusers+") | 7,485 | 8,943 |
| S3 | (MH "Alcoholism") OR (MH "Alcohol Drinking+") | 39,401 | 46,632 |
| S4 | (MH "Street Drugs+") | 5,313 | 6,091 |
| S5 | TI (alcohol OR drug* OR substance* OR "ill* drug*" OR "banned substance*" OR cannabi* OR marijuana OR cocaine OR heroin OR meth* OR "street drug*") N2 (use or misuse or abuse or intake or consum*) | 37,618 | 45,077 |
| S6 | AB (alcohol OR drug* OR substance* OR "ill* drug*" OR "banned substance*" OR cannabi* OR marijuana OR cocaine OR heroin OR meth* OR "street drug*") N2 (use or misuse or abuse or intake or consum*) | 91,085 | 116,201 |
| S7 | S1 OR S2 OR S3 OR S4 OR S5 OR S6 | 152,277 | 184,780 |
| S8 | TI prevent* OR promot* OR treat* OR manag* | 1,637,100 | 978,416 |
| S9 | AB prevent* OR promot* OR treat* OR manag* | 1,692,174 | 1,125,112 |
| S10 | S8 OR S9 | 1,737,401 | 1,184,751 |
| S11 | S7 AND S10 | 63,123 | 43,782 |
| S12 | (MH "Cost Benefit Analysis") | 29,391 | 35,296 |
| S13 | TI "Cost Benefit Analys?s" | 335 | 402 |
| S14 | AB "Cost Benefit Analys?s" | 754 | 945 |
| S15 | TI "economic evaluation*" | 2,141 | 2,507 |
| S16 | AB "economic evaluation*" | 3,004 | 3,776 |
| S17 | (MH "Costs and Cost Analysis") | 16,078 | 18,609 |
| S18 | TI "Cost Analys?s" | 1,011 | 1,211 |
| S19 | AB "Cost Analys?s" | 1,351 | 1,680 |
| S20 | TI "return on investment" | 276 | 319 |
| S21 | AB "return on investment" | 732 | 960 |
| S22 | TI "return to investment" | 276 | 319 |
| S23 | AB "return to investment" | 732 | 960 |
| S24 | TI "cost effectiveness analys?s" | 2,162 | 2,661 |
| S25 | AB "cost effectiveness analys?s" | 2,516 | 3,195 |
| S26 | TI "cost utility analys?s" | 489 | 589 |
| S27 | AB "cost utility analys?s" | 723 | 919 |
| S28 | S12 OR S13 OR S14 OR S15 OR S16 OR S17 OR S18 OR S19 OR S20 OR S21 OR S22 OR S23 OR S24 OR S25 OR S26 OR S27 | 49,389 | 58,755 |
| S29 | S11 AND S28 | 896 | 596 |
| S30 | S11 AND S28 (Limiters – English Language; Peer Reviewed; Human; Publication Type: Corrected Article, Journal Article) | 516 | 293 |
|  |  | 545 | |

Note: The highlighted term ‘treat*’ was not included in the 05/05/2021 to reflect the preventive focus of this review.

Search strategy (PsychInfo)

| # | Query | Results  (01/08/2019) | Results  (05/05/2021) |
| --- | --- | --- | --- |
| S1 | DE "Marijuana Usage" OR DE "Substance Use Disorder" OR DE "Addiction" OR DE "Alcohol Use Disorder" OR DE "Cannabis Use Disorder" OR DE "Drug Abuse" OR DE "Inhalant Abuse" OR DE "Opioid Use Disorder" OR DE "Tobacco Use Disorder" | 64,665 | 71,594 |
| S2 | DE "Alcohol Abuse" OR DE "Alcoholism" OR DE "Binge Drinking" OR DE "Alcohol Drinking Attitudes" OR DE "Alcohol Drinking Patterns" OR DE "Social Drinking" OR DE "Underage Drinking" OR DE "Alcohol Use Disorder" OR DE "Alcohol Abuse" OR DE "Alcohol Intoxication" | 73,544 | 78,203 |
| S3 | DE "Drug Abuse" OR DE "Inhalant Abuse" OR DE "Polydrug Abuse" OR DE "Drug Addiction" | 56,349 | 58,817 |
| S4 | TI (alcohol OR drug* OR substance* OR "ill* drug*" OR "banned substance*" OR cannabi* OR marijuana OR cocaine OR heroin OR meth* OR "street drug*") N2 (use or misuse or abuse or intake or consum*) | 52,612 | 58,367 |
| S5 | AB (alcohol OR drug* OR substance* OR "ill* drug*" OR "banned substance*" OR cannabi* OR marijuana OR cocaine OR heroin OR meth* OR "street drug*") N2 (use or misuse or abuse or intake or consum*) | 169,119 | 186,124 |
| S6 | S1 OR S2 OR S3 OR S4 OR S5 | 227,072 | 246,931 |
| S7 | TI prevent* OR promot* OR treat* OR manag* | 1,433,999 | 719,734 |
| S8 | AB prevent* OR promot* OR treat* OR manag* | 1,506,833 | 852,829 |
| S9 | S7 OR S8 | 1,510,452 | 859,085 |
| S10 | S6 AND S9 | 124,189 | 59,776 |
| S11 | TI "Cost Benefit Analys?s" | 190 | 201 |
| S12 | AB "Cost Benefit Analys?s" | 956 | 1,036 |
| S13 | TI "economic evaluation*" | 529 | 614 |
| S14 | AB "economic evaluation*" | 1,359 | 1,602 |
| S15 | DE "Costs and Cost Analysis" | 17,287 | 18,731 |
| S16 | TI "Cost Analys?s" | 192 | 208 |
| S17 | AB "Cost Analys?s" | 633 | 683 |
| S18 | TI "return on investment" | 109 | 129 |
| S19 | AB "return on investment" | 773 | 871 |
| S20 | TI "return to investment" | 109 | 129 |
| S21 | AB "return to investment" | 773 | 871 |
| S22 | TI "cost effectiveness analys?s" | 383 | 427 |
| S23 | AB "cost effectiveness analys?s" | 1,022 | 1,156 |
| S24 | TI "cost utility analys?s" | 75 | 85 |
| S25 | AB "cost utility analys?s" | 304 | 361 |
| S26 | S11 OR S12 OR S13 OR S14 OR S15 OR S16 OR S17 OR S18 OR S19 OR S20 OR S21 OR S22 OR S23 OR S24 OR S25 | 20,202 | 22,115 |
| S27 | S10 AND S26 | 1,092 | 554 |
| S28 | S10 AND S26 (Limiters – Peer Reviewed; Publication Type: Peer Reviewed Journal; English; Population Group: Human; Document Type: Erratum/Correction, Journal Article, Retraction) | 835 | 426 |
|  |  | 870 | |

Note: The highlighted term ‘treat*’ was not included in the 05/05/2021 to reflect the preventive focus of this review.

Search strategy (EconLit)

| # | Query | Results  (01/08/2019) | Results  (05/05/2021) |
| --- | --- | --- | --- |
| S1 | (ZW "substance use disorders") | 2 | 2 |
| S2 | (ZW "alcohol") or (ZW "alcohol availability, health, alcohol consumption") or (ZW "alcohol consumption") or (ZW "alcohol consumption, alcohol tax, binge drinking, beer, wine and spirits") or (ZW "alcohol misuse, morbidity, ordered probit, tobit, eq5d") or (ZW "alcohol, demand model, patterns of consumption") or (ZW "alcohol.") or (ZW "alcoholism") | 49 | 51 |
| S3 | (ZW "drug addiction") or (ZW "drug traffic") or (ZW "drugs") or (ZW "drugs of abuse") | 1,438 | 1,595 |
| S4 | TI (alcohol OR drug* OR substance* OR "ill* drug*" OR "banned substance*" OR cannabi* OR marijuana OR cocaine OR heroin OR meth* OR "street drug*") N2 (use or misuse or abuse or intake or consum*) | 1,021 | 1,084 |
| S5 | AB (alcohol OR drug* OR substance* OR "ill* drug*" OR "banned substance*" OR cannabi* OR marijuana OR cocaine OR heroin OR meth* OR "street drug*") N2 (use or misuse or abuse or intake or consum*) | 6,792 | 7,775 |
| S6 | S1 OR S2 OR S3 OR S4 OR S5 | 8,565 | 9,709 |
| S7 | TI prevent* or promot* or treat* or manag* | 341,062 | 333,678 |
| S8 | AB prevent* or promot* or treat* or manag* | 349,052 | 343,295 |
| S9 | S7 OR S8 | 349,822 | 344,159 |
| S10 | S6 AND S9 | 2,607 | 2,245 |
| S11 | (ZW "cost benefit") or (ZW "cost benefit analysis") | 887 | 1,001 |
| S12 | TI "Cost Benefit Analys?s" | 978 | 1,019 |
| S13 | AB "Cost Benefit Analys?s" | 1,751 | 1,885 |
| S14 | (ZW "economic evaluation") | 2 | 2 |
| S15 | TI "economic evaluation*" | 761 | 810 |
| S16 | AB "economic evaluation*" | 756 | 845 |
| S17 | (ZW "cost analysis") | 1 | 1 |
| S18 | TI "cost analys?s" | 590 | 627 |
| S19 | AB "cost analys?s" | 756 | 824 |
| S20 | (ZW "return on investment") | 3 | 9 |
| S21 | TI "return on investment" | 121 | 128 |
| S22 | AB "return on investment" | 599 | 682 |
| S23 | TI "return to investment" | 121 | 128 |
| S24 | AB "return to investment" | 599 | 682 |
| S25 | (ZW "cost effective") or (ZW "cost effectiveness") or (ZW "cost effectiveness analysis") | 527 | 623 |
| S26 | TI "cost effectiveness analys?s" | 307 | 328 |
| S27 | AB "cost effectiveness analys?s" | 412 | 453 |
| S28 | TI "cost utility analys?s" | 52 | 60 |
| S29 | AB "cost utility analys?s" | 98 | 118 |
| S30 | S11 OR S12 OR S13 OR S14 OR S15 OR S16 OR S17 OR S18 OR S19 OR S20 OR S21 OR S22 OR S23 OR S24 OR S25 OR S26 OR S27 OR S28 OR S29 | 6,460 | 7,002 |
| S31 | S10 AND S30 | 62 | 42 |
| S32 | S10 AND S30 (Limiters - Publication Type: Journal Article Search modes - Boolean/Phrase) | 50 | 34 |
|  |  | 55 | |

Note: The highlighted term ‘treat*’ was not included in the 05/05/2021 to reflect the preventive focus of this review.

| **Section and Topic** | **Item #** | **Checklist item** | **Reported (Yes/No)** |
| --- | --- | --- | --- |
| **TITLE** | | |  |
| Title | 1 | Identify the report as a systematic review. | Yes |
| **BACKGROUND** | | |  |
| Objectives | 2 | Provide an explicit statement of the main objective(s) or question(s) the review addresses. | Yes |
| **METHODS** | | |  |
| Eligibility criteria | 3 | Specify the inclusion and exclusion criteria for the review. | Yes |
| Information sources | 4 | Specify the information sources (e.g. databases, registers) used to identify studies and the date when each was last searched. | Yes |
| Risk of bias | 5 | Specify the methods used to assess risk of bias in the included studies. | Yes |
| Synthesis of results | 6 | Specify the methods used to present and synthesise results. | Yes |
| **RESULTS** | | |  |
| Included studies | 7 | Give the total number of included studies and participants and summarise relevant characteristics of studies. | Yes |
| Synthesis of results | 8 | Present results for main outcomes, preferably indicating the number of included studies and participants for each. If meta-analysis was done, report the summary estimate and confidence/credible interval. If comparing groups, indicate the direction of the effect (i.e. which group is favoured). | Yes |
| **DISCUSSION** | | |  |
| Limitations of evidence | 9 | Provide a brief summary of the limitations of the evidence included in the review (e.g. study risk of bias, inconsistency and imprecision). | Yes |
| Interpretation | 10 | Provide a general interpretation of the results and important implications. | Yes |
| **OTHER** | | |  |
| Funding | 11 | Specify the primary source of funding for the review. | No |
| Registration | 12 | Provide the register name and registration number. | Yes |

*From:*  Page MJ, McKenzie JE, Bossuyt PM, Boutron I, Hoffmann TC, Mulrow CD, et al. The PRISMA 2020 statement: an updated guideline for reporting systematic reviews. BMJ 2021;372:n71. doi: 10.1136/bmj.n71

For more information, visit: <http://www.prisma-statement.org/>

| **Section and Topic** | **Item #** | **Checklist item** | **Location where item is reported** |
| --- | --- | --- | --- |
| **TITLE** | | |  |
| Title | 1 | Identify the report as a systematic review. | Title (p. 1) |
| **ABSTRACT** | | |  |
| Abstract | 2 | See the PRISMA 2020 for Abstracts checklist. | Prisma 2020 for abstract (supp. file) |
| **INTRODUCTION** | | |  |
| Rationale | 3 | Describe the rationale for the review in the context of existing knowledge. | Introduction, Aims and Objectives (p. 3) |
| Objectives | 4 | Provide an explicit statement of the objective(s) or question(s) the review addresses. | Introduction, Aims and Objectives (p. 3) |
| **METHODS** | | |  |
| Eligibility criteria | 5 | Specify the inclusion and exclusion criteria for the review and how studies were grouped for the syntheses. | Methodology, Study selection (p. 4) and Table 1. |
| Information sources | 6 | Specify all databases, registers, websites, organisations, reference lists and other sources searched or consulted to identify studies. Specify the date when each source was last searched or consulted. | Methodology, Search strategy (p. 3-4) |
| Search strategy | 7 | Present the full search strategies for all databases, registers and websites, including any filters and limits used. | Search strategy (see Supplementary material) |
| Selection process | 8 | Specify the methods used to decide whether a study met the inclusion criteria of the review, including how many reviewers screened each record and each report retrieved, whether they worked independently, and if applicable, details of automation tools used in the process. | Methodology, Study selection (p. 4) |
| Data collection process | 9 | Specify the methods used to collect data from reports, including how many reviewers collected data from each report, whether they worked independently, any processes for obtaining or confirming data from study investigators, and if applicable, details of automation tools used in the process. | Methodology, Data extraction (p. 4) |
| Data items | 10a | List and define all outcomes for which data were sought. Specify whether all results that were compatible with each outcome domain in each study were sought (e.g. for all measures, time points, analyses), and if not, the methods used to decide which results to collect. | Methodology, Data extraction (p. 4) |
|  | 10b | List and define all other variables for which data were sought (e.g. participant and intervention characteristics, funding sources). Describe any assumptions made about any missing or unclear information. | Methodology, Data extraction (p. 4) |
| Study risk of bias assessment | 11 | Specify the methods used to assess risk of bias in the included studies, including details of the tool(s) used, how many reviewers assessed each study and whether they worked independently, and if applicable, details of automation tools used in the process. | Methodology, Quality assessment (p. 5-6) |
| Effect measures | 12 | Specify for each outcome the effect measure(s) (e.g. risk ratio, mean difference) used in the synthesis or presentation of results. | Methodology, Data extraction (p. 5) |
| Synthesis methods | 13a | Describe the processes used to decide which studies were eligible for each synthesis (e.g. tabulating the study intervention characteristics and comparing against the planned groups for each synthesis (item #5)). | Methodology, Data extraction (p. 5) |
|  | 13b | Describe any methods required to prepare the data for presentation or synthesis, such as handling of missing summary statistics, or data conversions. | Methodology, Data extraction (p. 5) |
|  | 13c | Describe any methods used to tabulate or visually display results of individual studies and syntheses. | Methodology, Finding synthesis (p. 5) |
|  | 13d | Describe any methods used to synthesize results and provide a rationale for the choice(s). If meta-analysis was performed, describe the model(s), method(s) to identify the presence and extent of statistical heterogeneity, and software package(s) used. | Methodology, Finding synthesis (p. 5) |
|  | 13e | Describe any methods used to explore possible causes of heterogeneity among study results (e.g. subgroup analysis, meta-regression). | N/A |
|  | 13f | Describe any sensitivity analyses conducted to assess robustness of the synthesized results. | N/A |
| Reporting bias assessment | 14 | Describe any methods used to assess risk of bias due to missing results in a synthesis (arising from reporting biases). | N/A |
| Certainty assessment | 15 | Describe any methods used to assess certainty (or confidence) in the body of evidence for an outcome. | N/A |
| **RESULTS** | | |  |
| Study selection | 16a | Describe the results of the search and selection process, from the number of records identified in the search to the number of studies included in the review, ideally using a flow diagram. | Results (p.6) |
|  | 16b | Cite studies that might appear to meet the inclusion criteria, but which were excluded, and explain why they were excluded. | Results (p.6) and Figure 1 |
| Study characteristics | 17 | Cite each included study and present its characteristics. | Results (p.6-9) and Table 2 |
| Risk of bias in studies | 18 | Present assessments of risk of bias for each included study. | Results (p.9) and Table 3 |
| Results of individual studies | 19 | For all outcomes, present, for each study: (a) summary statistics for each group (where appropriate) and (b) an effect estimate and its precision (e.g. confidence/credible interval), ideally using structured tables or plots. | Table 2 |
| Results of syntheses | 20a | For each synthesis, briefly summarise the characteristics and risk of bias among contributing studies. | Results, Colour grading of cost-effectiveness results (p.9-10) |
|  | 20b | Present results of all statistical syntheses conducted. If meta-analysis was done, present for each the summary estimate and its precision (e.g. confidence/credible interval) and measures of statistical heterogeneity. If comparing groups, describe the direction of the effect. | N/A |
|  | 20c | Present results of all investigations of possible causes of heterogeneity among study results. | N/A |
|  | 20d | Present results of all sensitivity analyses conducted to assess the robustness of the synthesized results. | N/A |
| Reporting biases | 21 | Present assessments of risk of bias due to missing results (arising from reporting biases) for each synthesis assessed. | N/A |
| Certainty of evidence | 22 | Present assessments of certainty (or confidence) in the body of evidence for each outcome assessed. | N/A |
| **DISCUSSION** | | |  |
| Discussion | 23a | Provide a general interpretation of the results in the context of other evidence. | Discussion (p.11-12) |
|  | 23b | Discuss any limitations of the evidence included in the review. | Discussion (p.11-12) |
|  | 23c | Discuss any limitations of the review processes used. | Discussion (p.11-12) |
|  | 23d | Discuss implications of the results for practice, policy, and future research. | Discussion (p.11-12) |
| **OTHER INFORMATION** | | |  |
| Registration and protocol | 24a | Provide registration information for the review, including register name and registration number, or state that the review was not registered. | Other information (p.14) |
|  | 24b | Indicate where the review protocol can be accessed, or state that a protocol was not prepared. | Other information (p.14) |
|  | 24c | Describe and explain any amendments to information provided at registration or in the protocol. | Other information (p.14) |
| Support | 25 | Describe sources of financial or non-financial support for the review, and the role of the funders or sponsors in the review. | Acknowledgments (p.14) |
| Competing interests | 26 | Declare any competing interests of review authors. | Declaration of interests (p.14) |
| Availability of data, code and other materials | 27 | Report which of the following are publicly available and where they can be found: template data collection forms; data extracted from included studies; data used for all analyses; analytic code; any other materials used in the review. | Data availability (p.14) |

*From:*  Page MJ, McKenzie JE, Bossuyt PM, Boutron I, Hoffmann TC, Mulrow CD, et al. The PRISMA 2020 statement: an updated guideline for reporting systematic reviews. BMJ 2021;372:n71. doi: 10.1136/bmj.n71

For more information, visit: <http://www.prisma-statement.org/>
